# Q‐LEAP: Millisecond Hyperdimensional Optimization for Full‐Spectrum Optical Metamaterials

**DOI:** 10.1002/advs.78032

**Published:** 2026-09-28

**Authors:** Zikang Guo, Yunwen Wu, Yuhui Guo, Shenghong Ju

**Affiliations:** ^1^ Department of Materials Science and Engineering Shanghai Jiao Tong University Shanghai China; ^2^ China‐UK Low Carbon College Shanghai Jiao Tong University Shanghai China; ^3^ Department of Engineering Mechanics Key Laboratory for Thermal Science and Power Engineering of Ministry of Education Tsinghua University Beijing China

**Keywords:** factorization machine, optical metamaterials, physics‑informed residual machine learning, quantum annealing, spectral properties calculation

## Abstract

Optical metamaterials exhibit superior optical performance, yet their high‐dimensional design problems impede efficient global optimization. In this study, we introduce Quantum‑driven Learning and Exploration for Advanced Photonic metamaterials (Q‐LEAP) that integrates material screening, property calculation and quantum‐accelerated structural design. By incorporating a factorization machine (FM) algorithm and leveraging the quantum tunnelling effect of quantum annealing, we efficiently explore a vast solution space of containing 2^108^ candidates, with a single optimization step completed in 2.56 ms, attaining an objective value that reaches 85.83% of its theoretical limit. To jointly evaluate runtime cost, sampling efficiency, and optimization effectiveness, we define normalized efficiency index on a natural‑log basis. Q‑LEAP reaches 21.77 × 10^−2^, approximately 40 times that of the best conventional method (0.55 × 10^−2^). Furthermore, we combine first‑principles calculations with a physics‑informed residual machine learning (PRML) method to predict frequency‑dependent dielectric functions. While density functional perturbation theory spectra are truncated at the phonon cutoff frequency, PRML overcomes this by using the Lorentz oscillator model as physical guidance, ensuring the high‑frequency extrapolation obeys the underlying oscillator dynamics. The Q‐LEAP constitutes an efficient design strategy for de novo optical metamaterials, with quantum‑driven optimization delivering a substantial speedup. This speedup can be transferred to high‑dimensional problems in other domains.

## Graphical abstract

Q‐LEAP integrates physics‐informed residual machine learning with factorization‐machine‐encoded quantum annealing to design full‐spectrum optical metamaterials. It explores a 2^108^ design space and, in a single 2.56 ms annealing step, reaches 85.83% of the theoretical FoM limit, enabling selective 5‐8 µm emission with 3–5 and 8–14 µm suppression and ∼40× higher normalized efficiency than conventional optimizers.

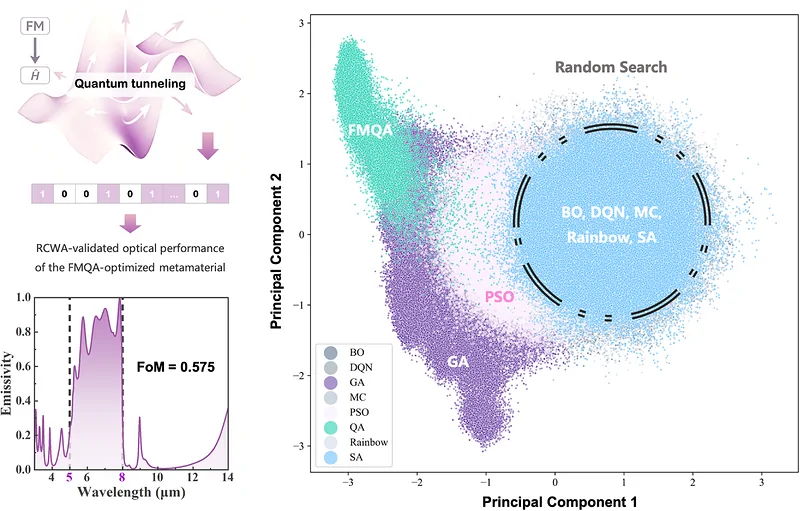

## 1 Introduction

As a disruptive artificially engineered electromagnetic material, optical metamaterials [1, 2, 3] are profoundly transforming cutting‐edge technologies such as optical interconnects [4, 5], super‐resolution microscopy [6], optical neural network computing [7], and quantum source manipulation [8]. Their core value lies in transcending the physical limits of natural materials in light‐field manipulation, enabling unprecedented applications through precise control over amplitude, phase, polarization, frequency spectrum, and other dimensions of light. For instance, Marcus Ossiander et al. [9] realize extreme ultraviolet metalenses by exploiting vacuum guiding in subwavelength holes perforated in a silicon membrane where the refractive index of silicon is less than unity, demonstrating focusing of 50‐nm‐wavelength light to a waist of 0.7 µm with a numerical aperture of 0.05. Aolong Sun et al. [10] propose an edge‐guided analog‐and‐digital optimization method to inversely design digital metamaterial‐based mode multiplexers, achieving a record multidimensional interconnect capacity of 38.2 Tb/s across 5 modes and 88 wavelength channels. However, deciphering the highly nonlinear and complex coupling mechanism between the electromagnetic response of materials and their macroscopic optical performance remains a critical bottleneck hindering the practical deployment of these advanced devices.

Inverse design of metamaterials has emerged as a research frontier, yet its high‐dimensional, strongly nonlinear nature renders conventional gradient‐based optimization inapplicable in most black‐box scenarios. To date, three categories of intelligent optimization strategies have been widely explored. Heuristic and metaheuristic algorithms, including genetic algorithms (GA), particle swarm optimization (PSO), simulated annealing (SA), and hybrid variants such as SA‐PSO [11], perform gradient‐free global search. For example, Sun et al. [12] combined GA with deep learning to achieve ultralow stiffness (∼0.188 nN/nm) and picogram‐level mass sensing (−0.801 kHz/pg), while Cao et al. [13] integrated GA with SA for inverse design of highly deformable mechanical metamaterials. However, although escape mechanisms like mutation or probabilistic acceptance are designed to avoid local optima, their effectiveness decays as iterations progress. Surrogate model‑based approaches, including Bayesian optimization with Gaussian process (GP) priors and deep learning‐based predictors, approximate structure‐response mappings with fewer evaluations. Xiao et al. [14] employed a conditional generative adversarial network (GAN) for thermal meta‑emitters, achieving solar reflectance > 0.96 and selectivity ratio up to 5.52. Zhu et al. [15] proposed a multi‐fidelity DeepONet for frequency transfer (relative error 0.043). Xu et al. [16] introduced a circuit‐level surrogate model for metacircuit‐embedded surfaces, generating six million samples on a laptop. Yet, GP‐based surrogates incur O(*N* ^3^) complexity (*N*: sample size), whose cost grows exponentially with dimension, while deep models demand large labeled datasets. Reinforcement learning (RL) has recently emerged as a promising paradigm. Yin et al. [17] developed MetaSeeker with population‑based RL, achieving 99.5% environmental similarity in dynamic cloaking with a 10^36^‐dimensional action space. Park et al. [18] proposed physics‑informed reinforcement learning (PIRL) that integrates adjoint gradients with deep Q‑learning, attaining > 90% diffraction efficiency in a 10^74^‐scale design space while improving sample efficiency by an order of magnitude over conventional RL. However, RL typically requires millions of training episodes, which is prohibitive in materials science where high‑fidelity data are inherently scarce. Consequently, existing methods face fundamental trade‑offs between global search capability, scalability, and data efficiency, motivating the development of a new optimization paradigm.

Among the most promising candidates is quantum computing, which offers fundamentally different search and sampling mechanisms. Quantum computing technologies can be broadly classified into two categories: universal quantum computing and special‐purpose quantum computing. Universal quantum computing encompasses various physical implementations, including superconducting qubits [19], trapped ions [20], neutral atoms, [21] and photonic qubits [22]. Special‐purpose quantum computing, on the other hand, is primarily represented by quantum annealing (QA) and coherent Ising machines, with D‑Wave's quantum annealers having reached commercial maturity. At present, universal quantum computers are constrained by the limited number of available logical qubits, rendering them incapable of performing large‐scale computational tasks. For instance, IBM's Nighthawk processor integrates three modules of 120 qubits each and is scheduled to demonstrate quantum advantage by 2026 [23]. By contrast, special‐purpose quantum annealers can support a substantially larger number of physical qubits, and their quantum acceleration is relatively easier to verify. D‑Wave Advantage system contains more than 5000 superconducting qubits, and its successor Advantage2, is equipped with over 4400 qubits [24]. In recent years, quantum annealing has been applied to materials science. King et al. [25] showed that a quantum annealer can outperform classical simulations of quantum spin glasses, matching its accuracy on the largest problems would take classical matrix product state methods millions of years on a supercomputer. Xu et al. [26] developed a quantum‑annealing‑assisted lattice optimization (QALO) algorithm for high‑entropy alloys, achieving superior mechanical properties while classical solvers (Gurobi, simulated annealing) fail to converge within 6 h. The core advantage of quantum annealing lies in the quantum tunnelling effect: particles can directly traverse energy barriers, thereby bypassing the thermal transition barriers inherent in classical simulated annealing. This mechanism enables quantum annealing to efficiently identify promising structures on a millisecond timescale.

To address the aforementioned limitations, we propose Quantum‑driven Learning and Exploration for Advanced Photonic metamaterials (Q‐LEAP), a framework that synergizes material screening, first‑principles property calculation, surrogate modelling via factorization machines, and quantum‑annealing‑based global optimization. Unlike conventional heuristic algorithms whose escape capability decays with iteration, Q‑LEAP leverages the quantum tunnelling effect inherent to quantum annealing to directly traverse energy barriers, fundamentally circumventing local entrapment and enabling millisecond‑scale exploration of a 2^108^ design space, a capability that remains challenging for classical simulated annealing or genetic algorithms within comparable time frames. Furthermore, Q‑LEAP leverages a factorization machine (FM) as a general‑purpose surrogate that learns the structure‐performance mapping from data and translates it into an Ising Hamiltonian, effectively bridging arbitrary physical objectives with quantum annealing hardware regardless of whether the original performance metric is energy‑related. This inherent adaptability of the FM‐based surrogate, which does not rely on any specific physical assumption, distinguishes Q‑LEAP from conventional forward‑model‑dependent frameworks. Crucially, to overcome the intrinsic spectral truncation of density functional perturbation theory (DFPT) at the phonon cutoff frequency, a bottleneck that has previously constrained first‑principles‑driven infrared design, we develop a physics‑informed residual machine learning (PRML) method. It incorporates a semi‑Lorentzian oscillator backbone as a physical constraint, enabling physically consistent extrapolation of dielectric functions into the target band while preserving the fitting fidelity of data‑driven models. The superiority of this quantum‑driven strategy over classical and learning‑based counterparts is quantitatively validated through comprehensive benchmarking against genetic algorithms, particle swarm optimization, simulated annealing, Bayesian optimization, and deep reinforcement learning models, e.g., the Deep Q‐Network (DQN) and Rainbow. Using a normalized efficiency index (NEI) that jointly accounts for solution quality (Figure of merit, FoM), convergence speed (Time to target, TTT), and sample efficiency (Evaluations to target, ETT), the proposed factorization machine quantum annealing (FMQA) framework consistently outperforms all baselines across all three metrics. Notably, once the FM surrogate is sufficiently trained on a large dataset, a single quantum annealing run suffices to yield a high‑quality solution, circumventing the iterative refitting and repeated sampling that conventional optimizers typically require. To our knowledge, this is the first demonstration of integrating quantum annealing with surrogate modelling and physics‑informed extrapolation for full‑spectrum optical metamaterial design, achieving 85.83% of the theoretical FoM limit with a single quantum annealing step of 2.56 ms.

## 2 Results

### 2.1 Q‐LEAP Framework

This study focuses on a Si/HfO_2_ multilayer metamaterial, consisting of a nine‐layer non‐periodic stack of alternating Si and HfO_2_ deposited on a SiO_2_ substrate. First, we retrieve all possible stable crystalline phases of Si, HfO_2,_ and SiO_2_ from crystallographic databases. Using DFPT, we accurately compute the frequency‑dependent dielectric functions of each material in the infrared range of 3–14 µm, thereby obtaining electromagnetic response parameters. Second, we construct a forward model based on rigorous coupled‑wave analysis (RCWA), which solves the vector wave equation for any given structure and yields its emission spectrum. Using random sampling, we generate 200 sets of structural parameters, with each layer thickness constrained within a process window of 10 nm to 36 µm. Optical properties in the target band are calculated for these 200 samples to build an initial dataset. An optimisation objective is then defined and mapped onto an Ising model via FM. The FM model is fed into a quantum annealer to perform large‑scale optimization, completing the structural design of the optical metamaterial. The Q‐LEAP described above is illustrated in Figure 1.

**FIGURE 1:**
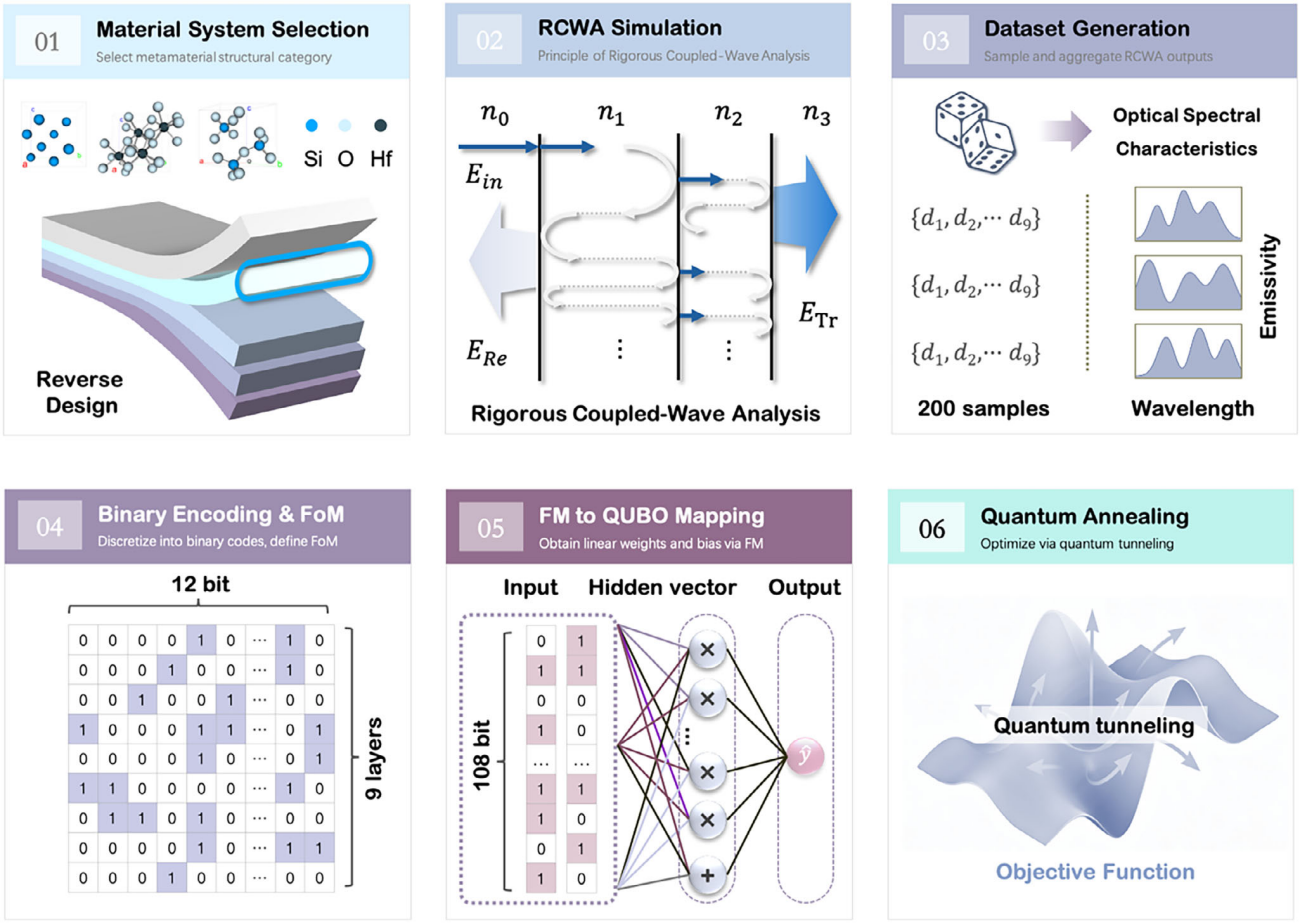
Q‐LEAP's framework for the design of optical metamaterials.

To quantify the target performance, a spectral selectivity FoM is defined in a general form as the difference between the average emissivity over a desired high‐emissivity band and the average emissivity over one or more low‐emissivity (suppression) bands:

(1) $$\begin{array}{c} \mathrm{FoM}\;=\sum_{m}\frac{1}{\Delta \lambda_{\mathrm{high},m}}\;\int_{\lambda_{\mathrm{high},m}^{\left(1\right)}}^{\lambda_{\mathrm{high},m}^{\left(2\right)}}\varepsilon \left(\lambda \right)d\lambda -\sum_{n}\frac{1}{\Delta \lambda_{\mathrm{low},n}}\int_{\lambda_{\mathrm{low},\;n}^{\left(1\right)}}^{\lambda_{\mathrm{low},n}^{\left(2\right)}}\varepsilon \left(\lambda \right)d\lambda \end{array}$$

In the present work, the high‐emissivity band is set to 5–8 µm (target emissivity *E*_5‐8_→1), while the suppression bands are 3–5 µm and 8–14 µm (*E*_3‐5,8‐14_→0). Substituting these specific wavelength intervals, the FoM simplifies to:

(2) $$\mathrm{FoM}\;=\frac{\int_{5}^{8}\varepsilon \left(\lambda \right)d\lambda }{3}\;-\frac{\int_{3}^{5}\varepsilon \left(\lambda \right)d\lambda }{2}-\frac{\int_{8}^{14}\varepsilon \left(\lambda \right)d\lambda }{6}$$

The 5–8 µm band is designed for high emission to facilitate radiative heat dissipation via atmospheric absorption, while the 3–5 and 8–14 µm windows are set at low emissivity to evade infrared detection, fulfilling the dual goals of cooling and stealth. The theoretical upper and lower limits of this FoM are 1 and ‐2, respectively.

After generating 200 sets of metamaterial structural parameters and calculating their corresponding FoM values via RCWA, we construct a surrogate model using FM. FM is a general‐purpose predictor that extends linear models by modelling all pairwise interactions between features through factorised latent vectors. It is particularly suited for high‐dimensional and sparse problems, where the number of training samples is limited relative to the feature space. Specifically, FM models the target as:

(3) $$\hat{y}=\omega_{0}+\sum_{i=1}^{N}\omega_{i}x_{i}+\sum_{1\leq i\leq j\leq N}\langle v_{i},\;v_{j}\rangle x_{i}x_{j}$$

where *x_i_* are the input features, ω_0_ and ω_*i*_ are global and linear weights, and *v_i_* is a *k*‐dimensional latent vector representing feature *i*. The inner product 〈*v_i_*, *v_j_* 〉 models the interaction between features *i* and *j*. In our case, the input is a 108‑dimensional feature vector, and the output is the predicted FoM. A key hyperparameter is the latent dimension *k*, which controls the expressiveness of the interaction terms.

To enable quantum optimization, the trained FM's weight parameters are mapped onto an Ising model, which naturally encodes combinatorial optimization problems as the ground‐state search of a spin Hamiltonian. The corresponding quadratic Hamiltonian takes the form:

(4) $$H\;\left(t\right)=\sum_{i=1}^{N}h_{i}^{z}\left(t\right)\sigma_{i}^{z}+\sum_{i\leq j}J_{ij}\left(t\right)\sigma_{i}^{z}\sigma_{j}^{z}$$

where the spin operator $\sigma_{i}^{z}$ corresponds to the film thickness design variable. Hardware embedding is performed on a quantum annealer using the Pegasus topology [27, 28, 29], with an annealing time of 20 µs, and low‑energy solutions are obtained by sampling. The optimized structure, obtained via quantum annealing sampling, is further validated through both local and global sensitivity analyses, confirming its robustness against thickness deviations within typical fabrication tolerances.

### 2.2 First‐Principles Calculation of Dielectric Spectra Over a Broad Frequency Range

The crystal structures of the three are all taken from the stable phases in the Materials Project [30] ‐single‐crystal Si (mp‐149, space group Fd‐3m), α‐quartz phase SiO_2_ (mp‐6930 and mp‐7000, space group P3_2_21), and monoclinic phase *m*‐HfO_2_ (mp‐352, space group P2_1_/c), whose primitive cell configurations are shown in Figure 2b, Notes S1 and S2. To obtain reliable dielectric function data, strict structural relaxation optimization is first performed within the framework of DFPT. The projector augmented wave (PAW) pseudopotential is used to describe the interaction between ionic cores and valence electrons. The plane‐wave cutoff energy is set to 520 eV to ensure energy convergence. The conjugate gradient algorithm is used in optimization process, with the convergence criteria of atomic force < 0.01 eV/Å and energy fluctuation < 1 × 10^−6^ eV.

**FIGURE 2:**
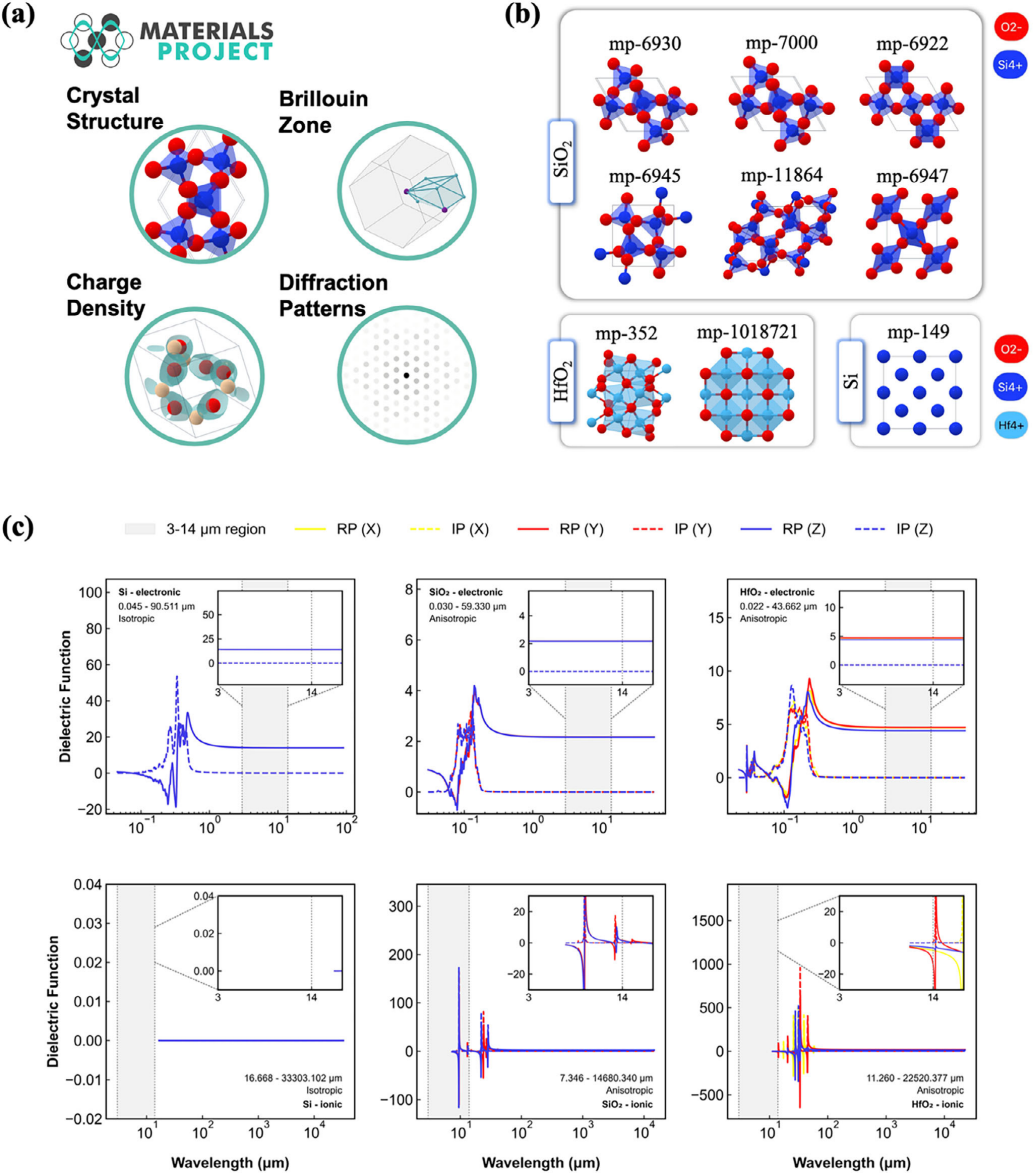
Crystal structure visualization and frequency‐dependent dielectric functions. (a) The nine crystal structures sourced from the Materials Project database. (b) Visualization of the core crystal structures (Si, SiO_2_, and HfO_2_) involved in this study. (c) Frequency‐dependent dielectric functions are shown in three columns for Si, SiO_2_, and HfO_2_. The top row showing electronic contributions, and the bottom row showing ionic contributions. The horizontal axes of all main panels and insets are in micrometers (µm). The main panels use a logarithmic scale to present the overall broadband trends, while the insets adopt a linear scale to clearly zoom in on the 3–14 µm spectral region of interest. In the legend, RP and IP denote the real and imaginary parts, respectively.

The dielectric response of single‑crystal silicon, shown in the first column of Figure 2c, reveals that, since its maximum phonon frequency corresponds to a wavelength of 16.668 µm and its ionic contribution remains identically zero across the broad spectral range from 16.668 µm to 33303.102 µm, only the electronic part contributes in the infrared region. For this electronic contribution to the dielectric function, the real part ε′ remains stable at 13.90, while the imaginary part ε′′ approaches zero. This behaviour originates from the symmetry of the diamond structure of silicon (space group Fd‐3m): all optical phonon modes at the Γ point are Raman‑active but infrared‑inactive. Group theoretical analysis shows that although the three optical branches are irreducible, the perfectly symmetric covalent bonds between Si atoms lead to cancellation of the dynamic dipole moments, resulting in zero infrared absorption cross‑section. Consequently, the ionic contribution vanishes, and in the infrared region far below the band‑gap energy (1.1 eV), the electronic contribution forms an almost constant dielectric plateau.

The results for the SiO_2_ system, with the electronic/ionic contribution presented in the second column of Figure 2c, exhibit a markedly different behavior. The electronic part varies slowly in the infrared range ($\varepsilon^{\prime }\;\approx \;2.25\;∼\;2.28$, ε′′  <  0.01), whereas the ionic contribution shows strong resonance features in the far‑infrared region. Specifically, we observe a main transverse‐optical (TO) mode peak at 450 cm^−1^ (corresponding to 22.222 µm) originating from Si‐O‐Si bending vibrations, and a high‐frequency shoulder at 1080 cm^−1^ (corresponding to 9.259 µm) arising from Si‐O stretching vibrations, indicating strong photon‐phonon coupling. Notably, although our target investigation band is set to 3–14 µm, the effective data range for the ionic contribution calculated via DFPT is inherently confined to 7.346–14 µm due to the physical limit imposed by the maximum phonon frequency of SiO_2_, approximately 40.839 THz (corresponding to 7.346 µm). At wavelengths shorter than this boundary, electromagnetic waves cannot effectively couple with the lattice phonons, causing the ionic polarization contribution to approach zero. SiO_2_ exhibits strong resonance only beyond 7.346 µm, making it ideal for precisely tailoring thermal radiation in that long‐wave infrared band.

The dielectric analysis of HfO_2_ reveals more complex behaviour, as shown in the third column of Figure 2c. The electronic part shows a monotonic decrease of ε′ from 4.40 to 4.39 over the 3–14 µm band, with ε′′ remaining below 0.005. In the low‑frequency region (> 14 µm), multiple resonance peaks appear in the ionic contribution. However, in the target band of 3–11.26 µm [167.40–627.66 (2π · THz)], the DFPT calculation suffers from a data gap (see Figure S3). This gap arises from a fundamental limitation of the computational method: DFPT‐based software solves the ionic dielectric function by numerical integration over discrete phonon frequencies. When the frequency exceeds the maximum phonon frequency ω_max_ of the system (167.40 2π · THz for *m*‑HfO_2_), the phonon density of states becomes zero, causing the denominator in the formula to diverge and preventing a valid solution. To fill this data gap, we have developed a physics‑informed residual machine learning algorithm. The results are detailed in Section 2.3.

To validate our calculated frequency‐dependent electronic, we compared the high‐frequency electronic contributions (reported in units of eV) with literature data. For single‑crystal silicon, our results show good agreement in the visible to near‑infrared range with the theoretical work of Lachenani et al. [31], who employed a plane‑wave pseudopotential method with the generalized gradient approximation‐Perdew‐Burke‐Ernzerhof (GGA‑PBE) functional, confirming the reliability of our approach, see Figure S2a. For silicon dioxide, our computed values deviate only slightly from the first‑principles study by Khattari et al. [32], which reported a static refractive index of 2.04 under ambient conditions, demonstrating the suitability of our model, see Figure S2b. For HfO_2_, the electronic contribution of our dielectric constants shows excellent agreement with the comprehensive DFT study by Mazumder et al. [33] at the key optical transition energy (∼10 eV), particularly in the peak position of the imaginary part, further validating our results, see Figure S2c. Regarding the ionic contribution, since DFPT cannot provide data beyond the phonon cutoff frequency, its comparison with experimental measurements is performed after extending the dielectric function using our PRML method, as elaborated in Section 2.3.

### 2.3 Physics‑Informed Residual Machine Learning for Dielectric Function Extrapolation

As noted in the previous subsection, the DFPT calculation for SiO_2_ and HfO_2_ suffers from a data gap in the target band of 3–14 µm. Specifically, the missing data for SiO_2_ cover the range from 3 to 7.346 µm, while for HfO_2_ the gap extends from 3 to 11.260 µm. In the high‑frequency regime beyond the phonon resonance, the ionic polarization contribution should smoothly decay to zero, as the ions cannot respond to the rapidly alternating external field due to their inertial mass. However, conventional DFPT cannot describe this decay trajectory, resulting in missing dielectric function data in a critical spectral range, which significantly restricts the design flexibility and necessitates experimental input for subsequent optical metamaterials.

To address the critical challenge that DFPT calculations for SiO_2_ and HfO_2_ cannot proceed beyond the maximum phonon frequency in the infrared band, we developed an innovative algorithm that integrates physical constraints with residual learning to overcome the dielectric‑function extrapolation bottleneck. Traditional methods suffer from significant limitations. First, we systematically evaluated six mainstream machine learning models: random forest (RF), decision tree (DT), gradient boosting (GB), K‑nearest neighbours (KNN), support vector regressor (SVR), and multilayer perceptron (MLP). As shown on the right side of Figure 3b, all these models exhibit excellent fitting performance on both training and test sets, with coefficients of determination consistently above 0.97. However, when extrapolated to high frequencies, every model failed catastrophically—the extrapolated results, provided in Figure S4, deviated severely from physical expectations, highlighting the inherent poor generalisation of purely data‑driven approaches. Second, we explored analytical models, constructing six functional forms: linear, quadratic, cubic, inverse‑proportional, exponential decay, and rational function (in linear fractional form), as shown on the left side of Figure 3b. The corresponding extrapolation performance of these models is shown in Figure S5. Among these, the linear fractional model emerged as the best analytical choice owing to its relatively strong extrapolation capability, yet its fitting accuracy in the high‑frequency region remained substantially lower than that of the machine learning models. This dilemma reveals the limitations of single‑method approaches: machine learning excels at high‑precision fitting but is weak at extrapolation, whereas analytical models possess extrapolation potential but lack fitting flexibility.

**FIGURE 3:**
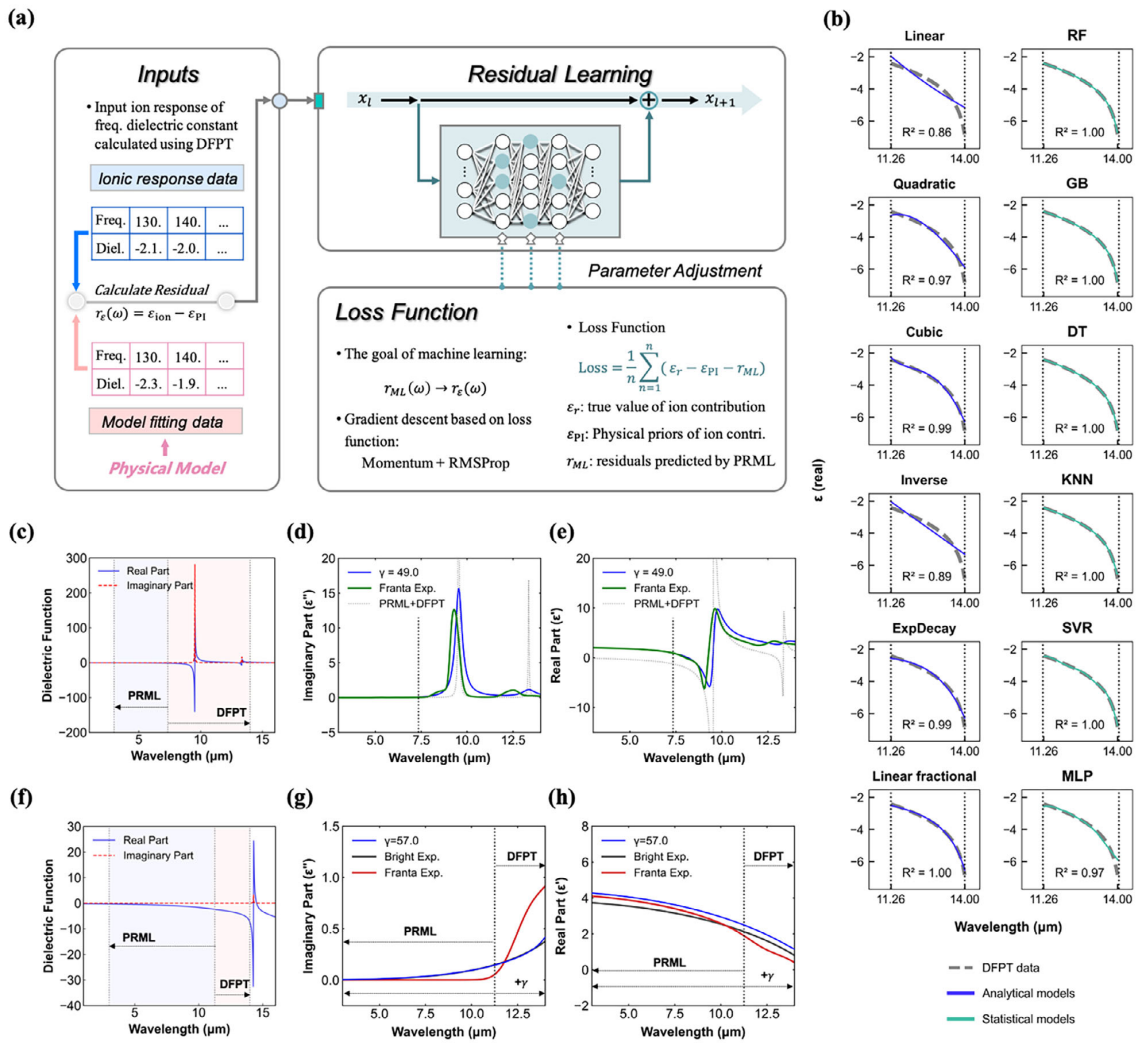
Performance evaluation of the physics‑informed residual machine learning model for dielectric function extrapolation. (a) Schematic of the physics‑informed residual machine learning framework. (b) Fitting performance of six analytical models and six statistical models against DFPT data for HfO_2_ in the 11.26–14.00 µm range. (c) Dielectric function completion for SiO_2_ in the 3–7.346 µm gap based on the PRML model. (d, e) Broadened spectra (damping coefficient *γ* = 49.0) from concatenated DFPT and PRML data for SiO_2_ over the 3–14 µm range, with (d) the imaginary part and (e) the real part, compared with experimental data from Franta et al. [34]. (f) Dielectric function completion for HfO_2_ in the 3–11.260 µm gap based on the PRML model. (g, h) Broadened spectra (damping coefficient *γ* = 57.0) from concatenated DFPT and PRML data for HfO_2_ over the 3–14 µm range, with (g) the imaginary part and (h) the real part, compared with experimental data from Franta et al. [35] and Bright et al. [36].

To combine the strengths of both paradigms, we propose a physics‑informed residual machine learning framework for fitting frequency‑dependent dielectric functions. Its backbone is the static ionic dielectric response derived from first principles:

(5) $$\varepsilon_{\mathrm{inoic}}=\frac{4\pi }{\Omega }\;\sum_{k}\frac{Z_{k}^{*}⊗Z_{k}^{*}}{\omega_{k}^{2}}$$

where $Z_{k}^{*}$ is the Born effective charge, ω_*k*_ the phonon frequency, and Ω the unit‑cell volume. This model, derived from first principles, encapsulates the fundamental physics of lattice dynamic responses and theoretically possesses excellent extrapolation behaviour. Our strategy treats the physical model as a strongly constrained backbone and introduces a residual learning mechanism. As depicted in the Figure 3a architecture, instead of directly predicting the dielectric constant, the ML model takes the ionic response inputs derived from DFPT alongside the physical priors ε_PI_. It specifically focuses on learning the complex residual deviation *r*_ML_(ω) between the theoretical predictions and the true first‐principles results. This predicted residual is then incorporated with the true value ε_*r*_ and the physical prior ε_PI_ to formulate the explicit loss function, which is optimized via gradient descent using momentum and root mean square propagation (RMSProp) for parameter adjustment, thereby precisely compensating for the deficiencies of the physical prior.

The validation results of the PRML demonstrate that it successfully integrates the extrapolation robustness of the physical model with the high‐precision fitting capability of machine learning. Within the training frequency range, the fitting accuracy of the proposed model surpasses that of any single analytical model and approaches the performance of pure machine learning. In the critical extrapolation region beyond the maximum phonon frequency, the model predictions exhibit excellent physical rationality, significantly outperforming all pure machine learning extrapolations that lack physical constraints, and effectively circumvent the typical issue of unconstrained divergence. Detailed model performance and relevant technical information are provided in Note S7.

Based on the results obtained via the PRML method, the predicted spectra for SiO_2_ and HfO_2_ were seamlessly combined with the DFPT bands, as shown in Figure 3c,f. It is observed that, with decreasing wavelength, the real part of the ionic contribution approaches zero in an exceptionally smooth manner. To verify the reliability of the model predictions in the spectral region exceeding the phonon cutoff frequency, we selected HfO_2_ as a representative case and compared our results with experimental data [35, 36] (see Note S8). A quantitative comparison between the dielectric function computed by the DFPT+PRML method and the experimental spectra reported by Franta [35] yielded mean squared errors (MSEs) of 0.024 for the refractive index *n* and 0.001 for the extinction coefficient *k*. Such remarkably low errors fully validate the reliability of our computational method within the target spectral range, thereby adequately supporting the subsequent optimization designs.

DFPT results show sharp peaks at specific frequencies that are incompatible with experimental spectra (Figure S7). This is because standard DFPT adopts the harmonic approximation with infinite phonon lifetimes. In reality, scattering mechanisms impart finite lifetimes and cause damping. Unlike this static undamped model proposed in Equation (5), the dynamic Lorentz oscillator model corrects the infinite‐lifetime assumption by introducing damping coefficients γ_*j*_, where the complex dielectric function is:

(6) $$\varepsilon \;\left(\omega \right)=\varepsilon_{\infty }+\sum_{j}\frac{S_{j}}{\omega_{0}^{2}-\omega^{2}-i\omega \gamma_{j}}$$

whose real and imaginary parts are:

(7) $$\varepsilon^{\prime }\;\left(\omega \right)=\varepsilon_{\infty }+\sum_{j}\frac{S_{j}\left(\omega_{j}^{2}-\omega^{2}\right)}{{\left(\omega_{j}^{2}-\omega^{2}\right)}^{2}+{\left(\gamma_{j}\omega \right)}^{2}}$$

(8) $$\varepsilon^{\prime \prime }\;\left(\omega \right)=\sum_{j}\frac{S_{j}\cdot \gamma_{j}\omega }{{\left(\omega_{j}^{2}-\omega^{2}\right)}^{2}+{\left(\gamma_{j}\omega \right)}^{2}}$$

Here, ***ω***_***j***_ and ***S***_***j***_ are derived from DFPT, while ***γ***_***j***_ is the introduced mode‐dependent broadening parameter. Because the macroscopic dielectric response is the linear superposition of all phonon contributions, simultaneous broadening of multiple peaks is required.

The damping coefficients ***γ***_***j***_ can be determined through ab initio molecular dynamics (AIMD) simulations, which naturally capture the anharmonic effects responsible for finite phonon lifetimes [36]. In this study, for the prominent peaks at 9.54 µm (SiO_2_) and 14.27 µm (HfO_2_), the extracted damping coefficients are γ  =  49 and γ  =  57, respectively. The resulting broadened spectra, presented in Figure 3d,e,g,h, exhibit excellent agreement with the experimental data, thereby confirming the reliability and accuracy of the adopted dynamic broadening framework. Further theoretical and computational details are provided in Note S9.

### 2.4 Hyperparameter Optimization of FM Model and Performance Comparison Between Quantum Annealing and Conventional Methods

For the optical metamaterial composed of nine alternating Si/HfO_2_ layers on a SiO_2_ substrate, with each layer thickness ranging from 0.01 µm to 36.00 µm encoded by 12‐bit binary strings, yielding a design space of dimension up to 2^108^, we first performed optimization tests using 200 initial Monte Carlo (MC) samples on the Quantum‐Inspired (QI) Annealer. When the sample size is very small, the factorization machine model suffers from severe underfitting due to data scarcity, so the Ising model can hardly capture material properties correctly. Consequently, quantum annealing optimization offers essentially no advantage over Monte Carlo search. Increasing the sample size is therefore necessary, and careful tuning of the factorization machine hyperparameters also provides an effective remedy.

The tuning of the key hyperparameter, the latent vector dimension *k*, is shown in Figure S10. A grid search over a broad parameter space with *k* ranging from 1 to 1500 revealed a nonmonotonic relationship between *k* and generalization performance. When *k* < 200, the training *R* ^2^ steadily rose to 0.78 with increasing *k*, while the test *R* ^2^ remained stable. For *k* > 200, the training *R* ^2^ saturated and the test *R* ^2^ declined markedly because of overfitting. Based on the Pareto front, we selected *k* = 10 as the optimal trade‐off between model complexity and prediction accuracy (see Figure S11).

Using the optimized factorization machine model, we performed 330 Ising optimization runs with QI‐Annealer and compared the results with those of 50 000 Monte Carlo searches, as shown in Figure S12a. Although the number of Monte Carlo searches exceeded that of the quantum‐inspired optimization by two orders of magnitude, its figure of merit values were predominantly concentrated around ‐0.5, and only very few samples reached FoM > 0. In contrast, the FoM of the samples from the quantum‐inspired Ising machine was mainly distributed around 0.5, clearly superior. We further employed two unsupervised dimensionality reduction methods, principal component analysis (PCA), and t‐distributed stochastic neighbour embedding (t‐SNE), to visually compare how the two sampling strategies populate the high‐dimensional design space. Unsupervised dimensionality reduction analyses further confirm that the quantum‑inspired sampler explores high‑performance regions of the design space that the Monte Carlo method struggles to access, and the detailed comparison between the two sampling strategies is provided in Figure S12.

To comprehensively validate the advantages of QA in both optimization performance and computational efficiency, we systematically compared it against a suite of classical and state‐of‐the‐art optimization methods. These include three heuristic algorithms (SA, GA, and PSO), one surrogate‐assisted method, Bayesian optimization (BO), and two deep reinforcement learning algorithms, DQN and Rainbow. The total number of RCWA calls was fixed at 200 000 for all algorithms except BO, which was constrained to 47 600 calls due to the exponential growth of computational complexity with problem dimensionality. To ensure statistical robustness and to mitigate the influence of initial samples and random seeds on optimization outcomes, multiple independent runs were performed for each algorithm. Specifically, SA was executed with 20 random seeds, each undergoing 10 000 generations. GA and PSO were also each executed with 20 random seeds, with a population size of 20 per seed and 500 generations per run, ensuring a total of 200 000 RCWA calls. For BO, given that Gaussian process regression is better suited for continuous variables, we adopted random forest (RF) and its variant, extra trees (ET), as surrogate models for the discrete design space, each with 10 random seeds. However, preliminary tests revealed that BO could sustain only approximately 2800 generations before encountering memory exhaustion. For DQN and Rainbow, 20 independent agents were deployed to explore the optimization space in parallel, with each agent iterating 10 000 steps. To maintain consistency with the physical objective of QA, minimizing the Ising Hamiltonian, the FoM values in Figure 4a–f were negated, thereby unifying the optimization objective across all algorithms as minimization. Each random seed was assigned to a single CPU core, and the total wall‐clock time for 20 parallel cores is annotated in the figure. As observed, GA achieves an optimal FoM of 0.530 after 200 000 iterations, whereas the FMQA attains a comparable FoM of 0.531 in merely 1365 iterations, demonstrating superior efficiency. Detailed parameter configurations, final optimization outcomes, and parallel runtime are summarized in Table 1.

**FIGURE 4:**
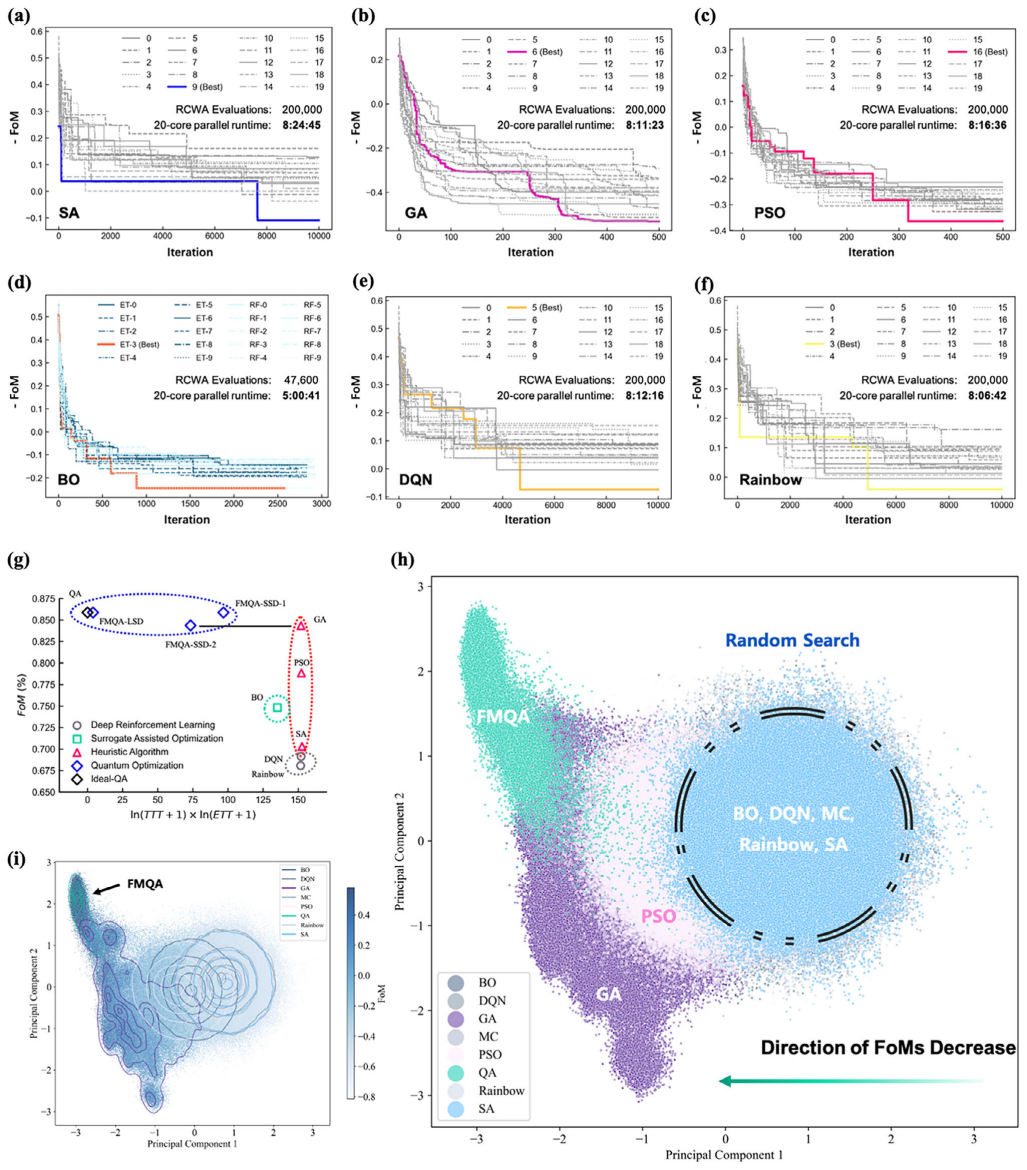
Performance comparison and design space distribution of different optimization algorithms. (a–f) Performance comparison of heuristic algorithms (SA, GA, PSO), surrogate‐assisted method (BO), and deep reinforcement learning (DQN and Rainbow) for metamaterial optimization. (g) Comprehensive performance comparison. Key labels are defined as follows. FMQA‐LSD denotes FMQA trained on a large‐scale dataset. FMQA‐SSD‐2 denotes FMQA trained on a small‐scale dataset that achieves a FoM comparable to that of GA. FMQA‐SSD‐1 denotes the small‐scale FMQA variant that yields the best FoM in the small‐scale dataset comparison, albeit with a relatively larger iteration budget. (h) PCA projection visualization of all collected samples. (i) Colormap encoding of the PCA projection based on sample FoM values.

**TABLE 1:** Parameter settings and performance comparison of optimization algorithms.

Category	Algorithm	RCWA Calls	Run time	Best FoM	Hyperparameters
Heuristic	SA	200 000	8:24:45	0.109	20 random seeds, 10 000 iterations per seed
	GA	200 000	8:11:23	0.530	20 seeds, population size = 20, 500 generations, crossover rate = 0.8, mutation rate = 0.01
	PSO	200 000	8:16:36	0.365	20 seeds, 20 particles, 500 iterations, *w* = 0.9, *c*_1_ = *c*_2_ = 1.0, *v*_max_ = 8.0
Surrogate Assisted	BO‐RF/ET	47 600	5:00:41	0.245	2 surrogate models, 10 random seeds per model, 10,000 sequential evaluations each
RL	DQN	200 000	8:12:16	0.074	20 independent agents, 10 000 steps each, *ε*‐greedy (1.0→0.01), buffer = 10k, batch = 32, *τ* = 0.005, MLP 108‐256‐256‐108, *lr* = 0.001
	Rainbow	200,000	8:06:42	0.042	20 agents, 10,000 steps each, Dueling + Double DQN + PER (*α* = 0.6) + *n*‐step (*n* = 3), same MLP, same *ε* decay and buffer as DQN

To establish a unified metric for algorithmic performance evaluation, we propose the Normalized Efficiency Index (NEI), which simultaneously accounts for algorithmic computational cost, sampling efficiency, and optimization effectiveness. NEI is defined as:

(9) $$\mathrm{NEI}=\frac{\mathrm{Fo}M_{\mathrm{achieve}}}{\mathrm{Fo}M_{\max}}\;\times \frac{1}{\ln\left(\mathrm{TTT}+1\right)}\times \frac{1}{\ln\left(\mathrm{ETT}+1\right)}$$

where *TTT* (Time to Target) denotes the computational time (in seconds) required to reach a specified FoM threshold, and *ETT* (Evaluations to Target) denotes the number of RCWA calls required to reach that threshold. Optimization effectiveness is quantified by the ratio of the achieved FoM to the theoretical maximum. The logarithmic transformation is introduced to compress the dynamic range of *TTT* and *ETT*, preventing variables with disproportionately large magnitudes (e.g., runtime in seconds) from dominating the index. The constant term “+1” is included to avoid taking the logarithm of zero and to keep the metric well‐defined for small *TTT*/*ETT*, such as FMQA‐LSD, which attains near‐optimal solutions within a single iteration, ensuring numerical stability of the metric.

Based on this definition, Figure 4g presents the comprehensive performance profile of all algorithms, with FoM (%) on the ordinate and **ln**(***TTT*** + 1) × **ln**(***ETT*** + 1) on the abscissa. The results reveal that FMQA‐based methods consistently achieve higher FoM with substantially reduced computational time. The logarithmic axis is used only to compress the dynamic range of *TTT* and *ETT* for display. For the present 108‐dimensional Si/HfO2 metamaterial design problem, the results demonstrate improved optimization/sampling efficiency relative to the tested classical baselines. Key labels in the figure are defined as follows. FMQA‐LSD (NEI = 21.77 × 10^−2^) denotes the FMQA model trained on a large‐scale dataset (LSD), achieving excellent solutions within a single iteration. FMQA‐SSD‐2 (NEI = 0.89 × 10^−2^) represents the FMQA model trained on a small‐scale dataset (SSD) that attains a FoM comparable to that of GA, with markedly fewer iterations and lower runtime. FMQA‐SSD‐1 (NEI = 1.15 × 10^−2^) also employs a small‐scale dataset but corresponds to the best FoM achieved in the small‐scale dataset comparison, at the cost of approximately 3401 iterations. Notably, the single‐iteration runtime of FMQA‐LSD is 295.0256 s, with the predominant overhead attributed to FM model training, while the QA inference process consumes only 2.56 ms. The leftmost black data point in the figure corresponds to QA without FM model training, which is applicable to problems directly mappable to Ising Hamiltonians and achieves the theoretical optimum in 2.56 ms, yielding the highest NEI (49.01), representing an ideal upper bound. For reference, the conventional heuristics (SA, GA, PSO), surrogate model‑based approaches (BO), and RL methods (DQN, Rainbow) exhibit NEI values of 0.46 × 10^−2^, 0.55 × 10^−2^, 0.52 × 10^−2^, 0.55 × 10^−2^, 0.45 × 10^−2^, and 0.45 × 10^−2^, respectively, all substantially lower than the FMQA variants.

To further elucidate the sampling distributions and search behaviors of different algorithms within the global design space, we performed principal component analysis on all collected samples (including the 50 000 samples generated during the Monte Carlo comparison) and visualized the results in a two‐dimensional projection, as shown in Figure 4h. The analysis reveals that BO is severely limited in high‐dimensional spaces due to its constrained iteration budget. DQN and Rainbow, while theoretically benefiting from large‐scale data, are hindered by the scarcity of training samples commonly encountered in materials design problems. SA tends to be trapped in local optima (despite its probabilistic acceptance of inferior solutions), thus failing to yield superior outcomes. In contrast, the population‐based GA and PSO exhibit significantly different distributions from Monte Carlo sampling in the PCA projection, indicating more directed exploration of the design space. Remarkably, the FMQA method successfully localizes the high‐performance region where the global optimum resides. Figure 4i further encodes the PCA projection with a colormap based on FoM values, where color intensity is correlated with FoM, revealing the clustering of high‐performance samples within the low‐dimensional latent space.

### 2.5 Quantum Annealing Optimization Results and RCWA Analysis

A typical quantum annealing workflow is depicted in Figure 5a, comprising three core stages: data preparation (Data), model fitting (Fit), and quantum annealing hardware optimization (Opt). The quantity and reliability of the data determine the predictive power of the model, while the accuracy of the resulting quadratic unconstrained binary optimization (QUBO) matrix directly influences the quality of the subsequent quantum annealing optimization. The new samples generated in the Opt stage are fed back into the dataset, thereby expanding the data volume. In materials science, where both time and computational cost are extremely expensive, the initial dataset is usually small (often fewer than 10^3^ samples). An iterative solving strategy therefore provides a more practical route to gradual optimization, and is more generally applicable. For problems where the dataset is already large, in principle a single quantum annealing run can yield a high‐quality solution, substantially reducing the cost of accessing a real quantum processing unit.

**FIGURE 5:**
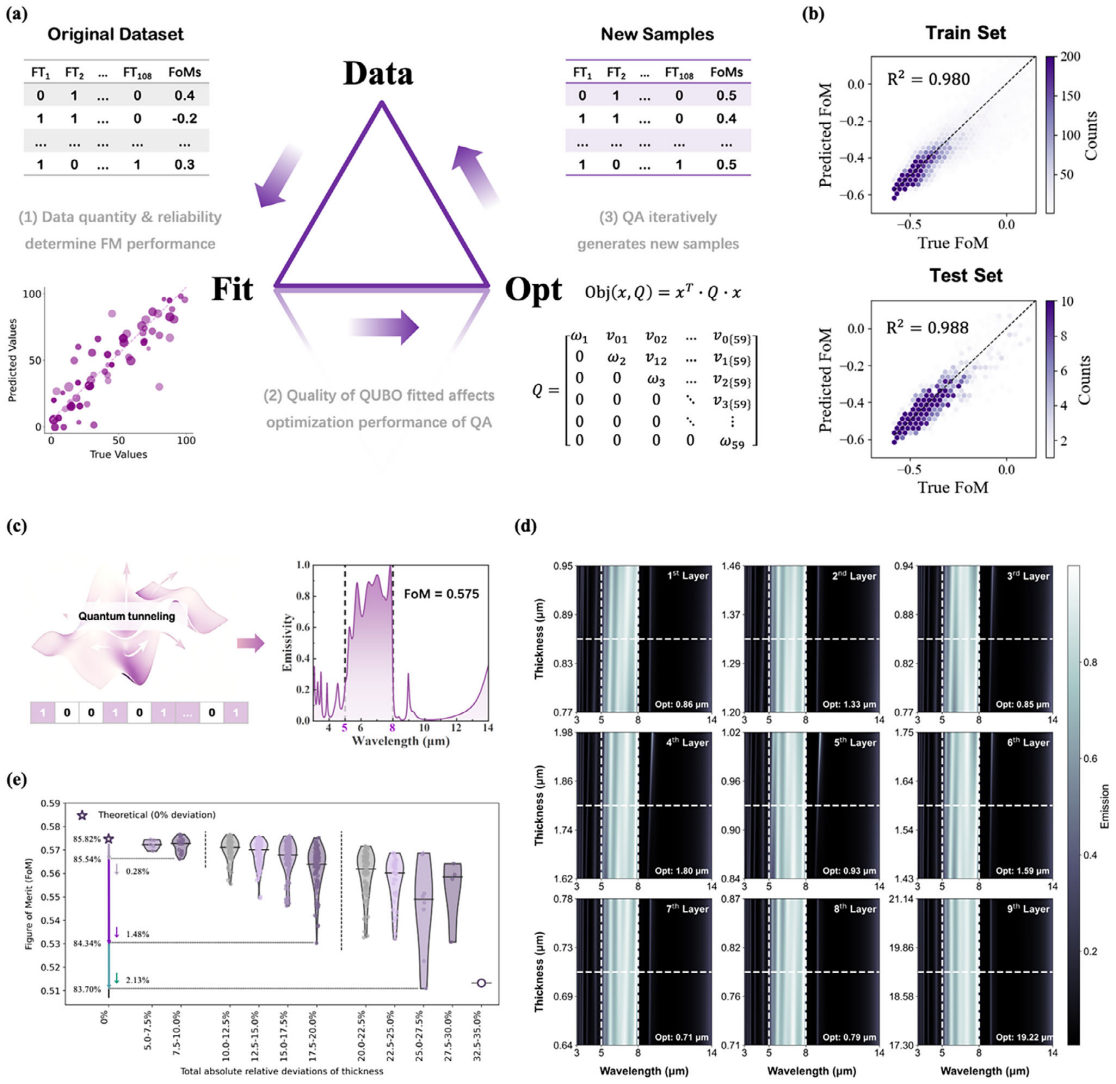
Summarizes the quantum annealing workflow, model training, optimization results, and sensitivity analysis. (a) The pipeline cycles through data assembly, model fitting, and quantum annealing optimization. Data quantity and QUBO matrix accuracy are critical. Optimized samples are fed back to expand the dataset. (b) The factorization machine model achieves an ***R*** ^2^ of 0.980 on the training set and 0.988 on the test set. (c) The optimal design obtained via quantum tunneling and its RCWA‑calculated emissivity are presented. (d) Local sensitivity analysis: emissivity maps for each of the nine layers under thickness perturbations within ±10% of the optimal value. White dashed lines mark the optimal thickness and the wavelength boundaries at 5 µm and 8 µm. (e) Global sensitivity analysis (Monte Carlo fabrication error analysis): all nine layers are simultaneously perturbed by normal random noise (*σ* = 2.5% for layers 1–8, *σ* = 1% for layer 9). Star marks the theoretical optimum (0% deviation). An open circle marks the only sample with deviation ≥ 30%.

Building on data accumulated from prior quantum‐inspired Ising machine tests and Monte Carlo searches, we trained an FM model on a large‐scale dataset of 102 000 samples (which covers only 3.143 × 10^−26^% of the entire solution space). Through hyperparameter tuning, the model achieves a coefficient of determination *R* ^2^> 0.980 on both the training and test sets. The hexbin plots of the training and test sets are shown in Figure 5b, confirming that the constructed Ising model faithfully captures the FoM of the optical metamaterial. A single quantum annealing optimization was then performed, yielding an FoM of 0.575 within a quantum processing unit (QPU) time of only 2.56 ms, reaching 85.83% of the theoretical limit. The optimized nine‑layer stack, in sequence from the incident side, is: Si (0.86 µm), HfO_2_ (1.33 µm), Si (0.85 µm), HfO_2_ (1.80 µm), Si (0.93 µm), HfO_2_ (1.59 µm), Si (0.71 µm), HfO_2_ (0.79 µm), and SiO_2_ (19.22 µm). The emissivity over the 3–14 µm range was validated by RCWA simulation, with the results visualized in Figure 5c: an average emissivity of 0.761 is achieved in the target band of 5–8 µm, while the emission in the stopbands of 3–5 µm and 8–14 µm is suppressed to 0.121 and 0.065, respectively. The emissivity in all critical stopbands remains below 0.13.

To comprehensively evaluate the impact of fabrication tolerances on the device performance, we first performed a local sensitivity analysis. Specifically, while keeping all other layers at their optimal thicknesses, we independently perturbed each of the nine layers by ±10% and calculated the full spectral emissivity (3–14 µm) for each perturbed case. The results are presented as a 3 × 3 colormap in Figure 5d, where each subplot corresponds to one layer, with the horizontal axis representing wavelength and the vertical axis representing the perturbed thickness. The white vertical dashed lines mark the two target wavelengths of 5 µm and 8 µm, while the white horizontal dashed line indicates the optimal thickness of that particular layer. As clearly observed, within the ±10% single‐layer perturbation range, the emissivity at both target wavelengths exhibits no dramatic fluctuations or sharp transitions, indicating that isolated thickness errors in a single layer have only a negligible impact on the spectral selectivity.

However, in actual micro‑ and nanofabrication processes (e.g., sputtering or electron‑beam evaporation), all layers are deposited or etched simultaneously rather than independently. Consequently, thickness errors inevitably occur concurrently across the entire multilayer stack, and the local analysis alone fails to capture the coupled systematic errors arising from simultaneous multi‑layer deviations. To address this, we conducted a more realistic global sensitivity analysis using a Monte Carlo approach with 500 random samples. Given that the first eight layers are relatively thin, we assigned them a normally distributed thickness error with a standard deviation of *σ*  =  2.5%. For the much thicker ninth SiO_2_ layer (19.22 µm), we assigned a more conservative error of *σ*  =  1%, considering that thicker films typically exhibit better thickness uniformity. It is worth noting that under this normal distribution setting, approximately 95% of the randomly sampled data fall within an overall relative deviation of ±5% (i.e., satisfying the statistical rule of 2*σ *≈ 5%). For each random sample, we computed the sum of absolute relative deviations across all nine layers (i.e., the cumulative absolute relative deviation) to quantify the overall fabrication error, and we plotted the corresponding FoM against this total deviation in Figure 5e. As shown in the figure, for cumulative absolute relative deviations of 10%, 20%, and 30%, the corresponding maximum performance degradations are only 0.28%, 1.48%, and 2.13%, respectively. These results demonstrate that even under the worst‑case superposition of multi‑layer errors, our optimized structure maintains excellent robustness and spectral stability within conventional fabrication tolerances, confirming the practical feasibility of the design.

## 3 Discussion

In summary, we have established the Q‐LEAP framework that seamlessly integrates materials screening from crystallographic databases, first‐principles dielectric property calculations, a physics‐informed residual machine learning algorithm to overcome the intrinsic phonon‐frequency limit of DFPT, surrogate modelling with factorization machines, and quantum annealing optimization. This closed‐loop pipeline autonomously converts a target spectral requirement into a structurally optimized metamaterial design. Leveraging a quantum annealer, we achieve efficient optimization over a solution space of 2^108^ candidate structures, using a training dataset that covers merely 3.143 × 10^−26^% of the total space. A single optimization step is completed in just 2.56 ms, and the obtained objective value reaches 85.83% of the theoretical limit, highlighting the substantial speedup and efficacy of quantum‐enabled global search. The resulting nine‐layer multilayer optical metamaterial delivers an average emissivity of 0.761 in the target 5–8 µm band, while the 3–5 µm and 8–14 µm suppression bands are suppressed to 0.121 and 0.065, respectively, demonstrating outstanding spectral selectivity. To rigorously benchmark the optimization performance, we systematically compared the FMQA against a suite of algorithms, including SA, GA, PSO, BO, DQN, and Rainbow, under identical RCWA evaluation budgets. The results demonstrate that FMQA‐based methods consistently achieve superior FoM values with dramatically fewer iterations and substantially lower computational cost, confirming the marked advantages of the quantum‐assisted surrogate approach in both optimization efficacy and sampling efficiency. Beyond algorithmic performance, we further assessed the practical feasibility of the optimized design against inevitable fabrication imperfections through sensitivity analyses. Local sensitivity analysis, performed by independently perturbing each of the nine layers by ±10%, reveals that isolated thickness errors induce negligible emissivity fluctuations at the target wavelengths of 5 µm and 8 µm. Also, we conducted a global sensitivity analysis with 500 random samples, where the first eight layers were assigned normally distributed errors with *σ* = 2.5% and the ninth SiO_2_ layer with *σ* = 1%. Quantifying the cumulative fabrication error as the sum of absolute relative deviations across all nine layers, we find that the maximum performance degradation is only 0.28% when the deviation is within 10%. As it reaches 20% and 30%, the degradation increases to 1.48% and 2.13%, respectively. These results confirm that the optimized structure maintains excellent spectral robustness within conventional fabrication tolerances. Crucially, the framework's generality is rooted in the PRML method that bypasses the DFPT extrapolation bottleneck, a discrete encoding that maps structures onto binary variables suitable for quantum annealing, and a hybrid classical–quantum computing strategy that orchestrates these components. These features make the approach directly transferable to other photonic, phononic, and mechanical metamaterials, offering a new paradigm for accelerating the inverse design of complex functional structures.

## 4 Methods

### 4.1 First‐Principles Calculations of Broadband Frequency‐Dependent Dielectric Spectra

In a physically periodic system, the Fourier transform of the frequency‐dependent symmetric microscopic dielectric matrix within the random‐phase approximation is given by:

(10) $$\varepsilon_{G,G^{\prime }}\;\left(q,\omega \right)=\delta_{G,G^{\prime }}\;-\frac{4\pi e^{2}}{\left|G+q\right|\left|G^{\prime }+q\right|}\chi_{G,G^{\prime }}^{0}\left(q,\omega \right)$$

where **G** and **G′** are reciprocal lattice vectors, and **q** denotes the Bloch wavevector of the incident wave. The matrix ***χ*** ^0^(**q**, ***ω***) is the irreducible polarizability in the independent‐particle picture, derived by Adler and Wiser within the self‐consistent field framework. The independent‐particle polarizability (Adler–Wiser formula) reads:

(11) $$\begin{array}{cc} \chi_{G,G^{\prime }}^{0}\;\left(q,\omega \right) & =\frac{1}{\Omega }\;\sum_{n,n^{\prime },k}2w_{k}\left(f_{n^{\prime }k+q}-f_{\mathrm{nk}}\right)\\ & \;\times \frac{\langle \psi_{n^{\prime }k+q}\left|e^{i\left(q+G\right)r}\right|\psi_{\mathrm{nk}}\rangle \langle \psi_{\mathrm{nk}}\left|e^{-i\left(q+G^{\prime }\right)r^{\prime }}\right|\psi_{n^{\prime }k+q}\rangle }{\varepsilon_{n^{\prime }k+q}-\varepsilon_{\mathrm{nk}}-\omega -i\eta } \end{array}$$

This is a central result of linear‐response theory, describing the ability of the electron system to polarize under a perturbation of frequency ***ω*** and wavevector q. Here ***Ω*** is the unit‐cell volume, and the k‐point weights ***w***_**k**_ satisfy a normalization condition. At zero temperature, the Fermi–Dirac distribution ***f***_***n*** **k**_ reduces to a step function, meaning ***f***_***n*** **k**_ =  1 for occupied states and 0 for unoccupied states. The factor of 2 accounts for spin degeneracy.

Using the projector augmented wave, the imaginary part of the dielectric function is simplified to:

(12) $$\begin{array}{cc} \varepsilon_{\infty }^{\left(2\right)}\;\left(\hat{q},\omega \right) & =\frac{4\pi^{2}e^{2}}{\Omega }\;\lim_{q\to 0}\frac{1}{{\left|q\right|}^{2}}\sum_{c,v,k}2w_{k}\delta \left(\varepsilon_{ck+q}-\varepsilon_{vk}-\omega \right)\\ & \;\times {\left|\langle u_{ck+q}|u_{vk}\rangle \right|}^{2} \end{array}$$

where indices *c* and *v* label conduction‐band and valence‐band states, respectively. Extending the equation to anisotropic materials, the dielectric constant becomes a 3 × 3 tensor:

(13) $$\begin{array}{cc} \varepsilon_{\alpha \beta }^{\left(2\right)}\;\left(\omega \right) & =\frac{4\pi^{2}e^{2}}{\Omega }\;\lim_{q\to 0}\frac{1}{q^{2}}\sum_{c,v,k}2w_{k}\delta \left(\varepsilon_{ck}-\varepsilon_{vk}-\omega \right)\\ & \;\times \langle u_{ck+e_{\alpha }q}|u_{vk}\rangle {\langle u_{ck+e_{\beta }q}|u_{vk}\rangle }^{*} \end{array}$$

Here ***e***_***α***_ and ***e***_***β***_ are unit vectors along the Cartesian directions. This expression allows calculation of the anisotropic optical response for light polarized along different crystal directions. The real part of the dielectric function is then obtained via the Kramers–Kronig transformation:

(14) $$\varepsilon_{\alpha \beta }^{\left(1\right)}\;\left(\omega \right)=1+\frac{2}{\pi }P\int_{0}^{\infty }\frac{\varepsilon_{\alpha \beta }^{\left(2\right)}\left(\omega^{\prime }\right)\omega^{\prime }}{{\left(\omega^{\prime }\right)}^{2}-\omega^{2}}d\omega^{\prime }$$

where P denotes the Cauchy principal value, reflecting the principle of physical causality. This relationship implies that the absorptive behaviour (imaginary part) at all frequencies determines the refractive response (real part) at any given frequency. Equations (13) and (14) constitute the working formulae for computing frequency‐dependent dielectric functions.

### 4.2 Transfer Matrix Method for Multilayer Structures

For a one‐dimensional optical metamaterial consisting of *q* uniform thin‐film layers stacked along the z‐direction, the optical properties of each layer are characterized by its complex refractive index  ***N***_***r***_ = ***n***_***r***_  − ***iκ***_***r***_, physical thickness ***d***_***r***_, and the propagation angle ***θ***_***r***_(determined recursively via Snell's law). In the transfer matrix method, each individual layer is represented by a 2×2 characteristic matrix:

(15) $$M_{1}=\left[\begin{array}{cc} \cos\delta_{r} & i\sin\delta_{r}\;/\;\eta_{r}\\ i\eta_{r}\sin\delta_{r} & \cos\delta_{r} \end{array}\right]\;$$

where ***δ***_**r**_ = 2 ***πN***_**r**_ ***d***_**r**_cos ϑ_**r**_/***λ***  is the phase thickness of the *r*‐th layer, with *λ* being the vacuum wavelength. The total characteristic matrix of the entire multilayer system is obtained by multiplying the individual layer matrices in the order of light propagation (from the incident side to the substrate):

(16) $$M_{\mathrm{total}}=M_{q}\;M_{q-1}\text{…}M_{2}M_{1}$$

Let ***η***_***m***_ be the optical admittance of the substrate and ***η***_0_ that of the incident medium. Applying the total characteristic matrix to the normalized electromagnetic field vector at the substrate interface yields the normalized electric field *B* and normalized magnetic field *C* at the incident interface:

(17) $$\left[\begin{array}{c} B\\ C \end{array}\right]=M_{\mathrm{total}}\;\left[\begin{array}{c} 1\\ \eta_{m} \end{array}\right]=\left\{\prod_{r=1}^{q}\left[\begin{array}{cc} \cos\delta_{r} & \frac{i\sin\delta_{r}}{\eta_{r}}\\ i\eta_{r}\sin\delta_{r} & \cos\delta_{r} \end{array}\right]\right\}\;\left[\begin{array}{c} 1\\ \eta_{m} \end{array}\right]$$

where ***η***_***r***_ is the tilted optical admittance (given by ***η***_***r***_ = ***N***_***r***_ cos ϑ_***r***_ for TE polarization and ***η***_***r***_ = ***N***_***r***_ /cos ϑ_***r***_ for TM polarization), and ***η***_***m***_ is the admittance of the substrate. The reflectance *R*, transmittance *T*, and absorptance *A* of the multilayer structure are given by:

(18) $$R=\left(\frac{\eta_{0}B-C\;}{\eta_{0}B+C}\right)\;{\left(\frac{\eta_{0}B-C\;}{\eta_{0}B+C}\right)}^{*}$$

(19) T=4η0Reηmη0B+Cη0B+C∗

(20) A=1−R−T

Equations (15)–(20) constitute the theoretical foundation for analyzing the reflection, transmission, and absorption spectra of arbitrary one‐dimensional multilayer structures, including optical metamaterials. For strictly periodic structures, the total characteristic matrix can be expressed as the *p*‐th power of a single‐period matrix, which facilitates further analysis of the photonic band structure.

## Author Contributions

S. Ju and Y. Wu jointly conceived the research idea, supervised the project, and provided critical guidance on the methodology design. Z. Guo designed the overall research framework, implemented all codes, performed data processing and visualization, and wrote the drafts of the manuscript. Y. Guo performed the systematic literature review and reference organization, and prepared the figures. All authors participated in revising the manuscript and approved the final version.

## Conflicts of Interest

The authors declare no conflicts of interest.

## Supporting information




**Supporting File**: advs78032‐sup‐0001‐SuppMat.pdf.

## Data Availability

The data that support the findings of this study are available from the corresponding author upon reasonable request.
