# Supplementary material for: Q‐LEAP: Millisecond Hyperdimensional Optimization for Full‐Spectrum Optical Metamaterials
Source: Adv Sci (Weinh). 2026 Sep 28:e78032. Online ahead of print. doi: 10.1002/advs.78032 (PMC13620267; doi:10.1002/advs.78032)
Supplement: Supplementary file 1 [file ADVS-9999-e78032-s001.pdf]

# Supporting Information

**Q-LEAP: Millisecond Hyperdimensional Optimization for Full-Spectrum Optical Metamaterials**

*Zikang Guo[1], Yunwen Wu[1*], Yuhui Guo[2], Shenghong Ju[2,3*]*

[1] Department of Materials Science and Engineering, Shanghai Jiao Tong University, Shanghai, China

[2] China-UK Low Carbon College, Shanghai Jiao Tong University, Shanghai, China

[3] Key Laboratory for Thermal Science and Power Engineering of Ministry of Education, Department of Engineering Mechanics, Tsinghua University, Beijing, China

[*] Corresponding Emails: tlwuyunwen@sjtu.edu.cn (Y. Wu), jush@tsinghua.edu.cn (S. Ju)

# Supplementary Note

## Supplementary Note 1 | Benchmark dielectric materials for frequency-dependent dielectric function calculations

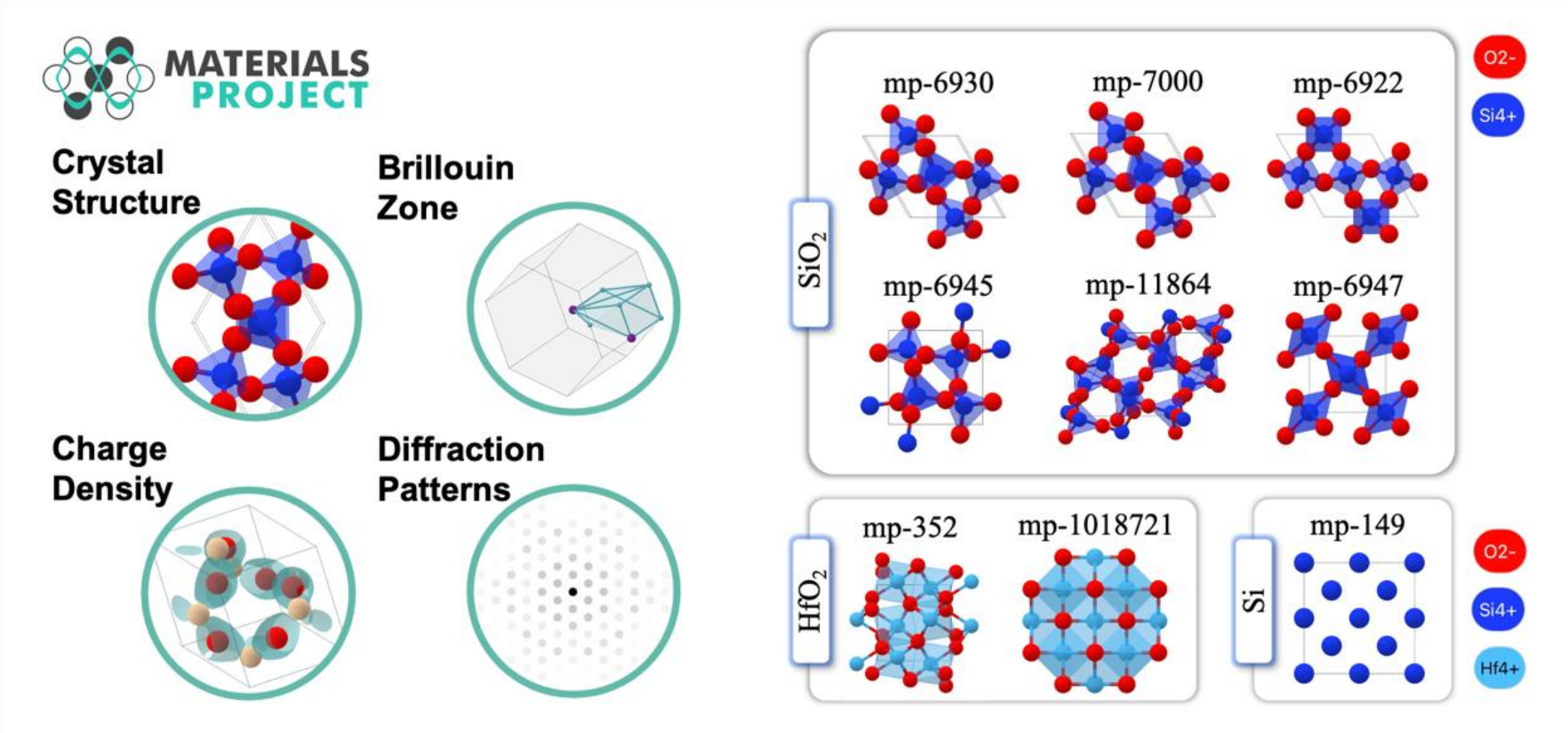


**FiGURE S1** | The nine crystal structures sourced from the Materials Project database

To compute the frequency-dependent dielectric function, this study first selected several conventional dielectric materials-$SiO_2$, $HfO_2$, and Si-as benchmark systems, and compared the results with existing theoretical calculations and experimental data. These materials are easy to prepare experimentally, facilitating experimental validation in practical applications such as photonic crystal optimisation. Moreover, literature data provide a reliable basis for assessing the accuracy of the PAW-based dielectric function calculations. The stable crystal structures and fundamental dielectric properties of the three materials were surveyed to establish a robust crystallographic and physical parameter foundation for subsequent frequency-dependent dielectric calculations.

Single-crystal silicon, used as a reference system, adopts the diamond structure ($Fd\bar{3}m1$) and exhibits typical covalent bonding characteristics. Its static dielectric constant has zero ionic contribution and an electronic contribution of 13.90, consistent with the absence of infrared-active optical phonon modes.

For $SiO_2$, five typical crystalline phases were examined. $\alpha$-quartz (space group $P3_121$ or $P3_221$), the most stable phase at ambient conditions, shows moderate dielectric anisotropy, with an ionic contribution of 2.20 and an electronic contribution of 2.54 to the static dielectric constant. $\beta$-quartz ($P6_222/P6_422$), stable at high temperatures, has a slightly lower ionic contribution of 2.17 and an electronic contribution of 2.35, reflecting the effect of increased structural symmetry on polarisation behaviour. $\alpha$-cristobalite ($P4_1\bar{2}_1\bar{2}$), with a more open structure, exhibits a significantly reduced ionic contribution of 1.80 and an electronic contribution of 2.24. In addition, the high-pressure phases coesite

(C12/c1) and stishovite ($P4_2/mnm$) were considered. Although complete dielectric data are not available for these phases, they are important for understanding the high-pressure behaviour of $SiO_2$.

For the $HfO_2$ system, the room-temperature stable monoclinic phase *m*-$HfO_2$ ($P12_1/c1$) and the high-temperature stable tetragonal phase *t*-$HfO_2$ ($P4_2/nmc$) were investigated. *m*-$HfO_2$ exhibits a high static dielectric constant, with an ionic contribution of 13.94 and an electronic contribution of 4.81, which is closely related to its pronounced soft optical phonon modes. *t*-$HfO_2$, stable above 1700 K, is not listed in the current database for its dielectric constant, but is known to potentially exhibit ferroelectric behaviour due to its non-centrosymmetric structure. Through doping, it can be stabilised at room temperature and holds promise for use in advanced electronic devices.

All crystal structures considered were taken from the Materials Project database [1,2] and are illustrated in **Figure S1**. The detailed stability conditions, space group symmetries and Materials Project IDs are summarised in **Table S1** ($SiO_2$) and **2** ($HfO_2$ and Si). An assessment of the importance of each crystalline phase for both fundamental research and technological applications is also provided in the tables.

## Supplementary Note 2 | Summary of structural parameters, stability conditions of the selected $SiO_2$, $HfO_2$ and Si polymorphs

**Table S1 | Structural parameters, stability conditions, and functional importance of $SiO_2$ polymorphs**

| Phase | Stability Conditions | Importance | Space group | mp-id |
|---|---|---|---|---|
| ***α*-quartz** | Ambient conditions | Most stable $SiO_2$ polymorph at room temperature and pressure | $P3_121/P3_221$ | mp-6930 |
| ***β*-quartz** | Ambient pressure, >573 °C | Key to understanding quartz phase transitions and high-temperature behaviour | $P6_222/P6_422$ | mp-6922 |
| ***α*-cristobalite** | Ambient pressure, ~1470 °C-1723 °C | Found in volcanic rocks, fired silica bricks and some biogenic siliceous structures | $P4_1\bar{2}_1\bar{2}$ | mp-6945 |
| **Coesite** | >2–3 GPa, wide temperature range | Indicator mineral for meteorite impact craters and ultrahigh-pressure metamorphic rocks | C12/c1 | mp-11684 |
| **Stishovite** | >8–10 GPa, wide temperature range | Diagnostic mineral of meteorite impact craters | $P4_2/mnm$ | mp-6947 |

**Table S2 | Structural parameters, stability conditions, and functional importance of $HfO_2$ and Si**

| Phase | Stability Conditions | Importance | Space group | mp-id |
|---|---|---|---|---|
| ***m*-$HfO_2$** | Ambient conditions | Addresses gate leakage current in advanced CMOS devices | $P12_1/c1$ | mp-352 |
| ***t*-$HfO_2$** | Ambient pressure, >1700 K | Parent structure for ferroelectric hafnia; can be stabilised at room temperature by doping | $P4_2/nmc$ | mp-1018721 |
| **Si** | Ambient conditions | Fundamental material for the semiconductor industry, with perfect lattice structure and controllable properties | $Fd\bar{3}m1$ | mp-149 |

This note compiles the detailed crystallographic data, stability regimes, Materials Project identifiers, and a brief assessment of the technical importance for each crystalline phase of $SiO_2$ (**Table S1**) and of $HfO_2$ and Si (**Table S2**). These data provide the structural and physical parameter basis for the frequency-dependent dielectric function calculations discussed in **Supplementary Note 1**.

## Supplementary Note 3 | Validation of calculated electronic contributions to the dielectric function against literature benchmarks

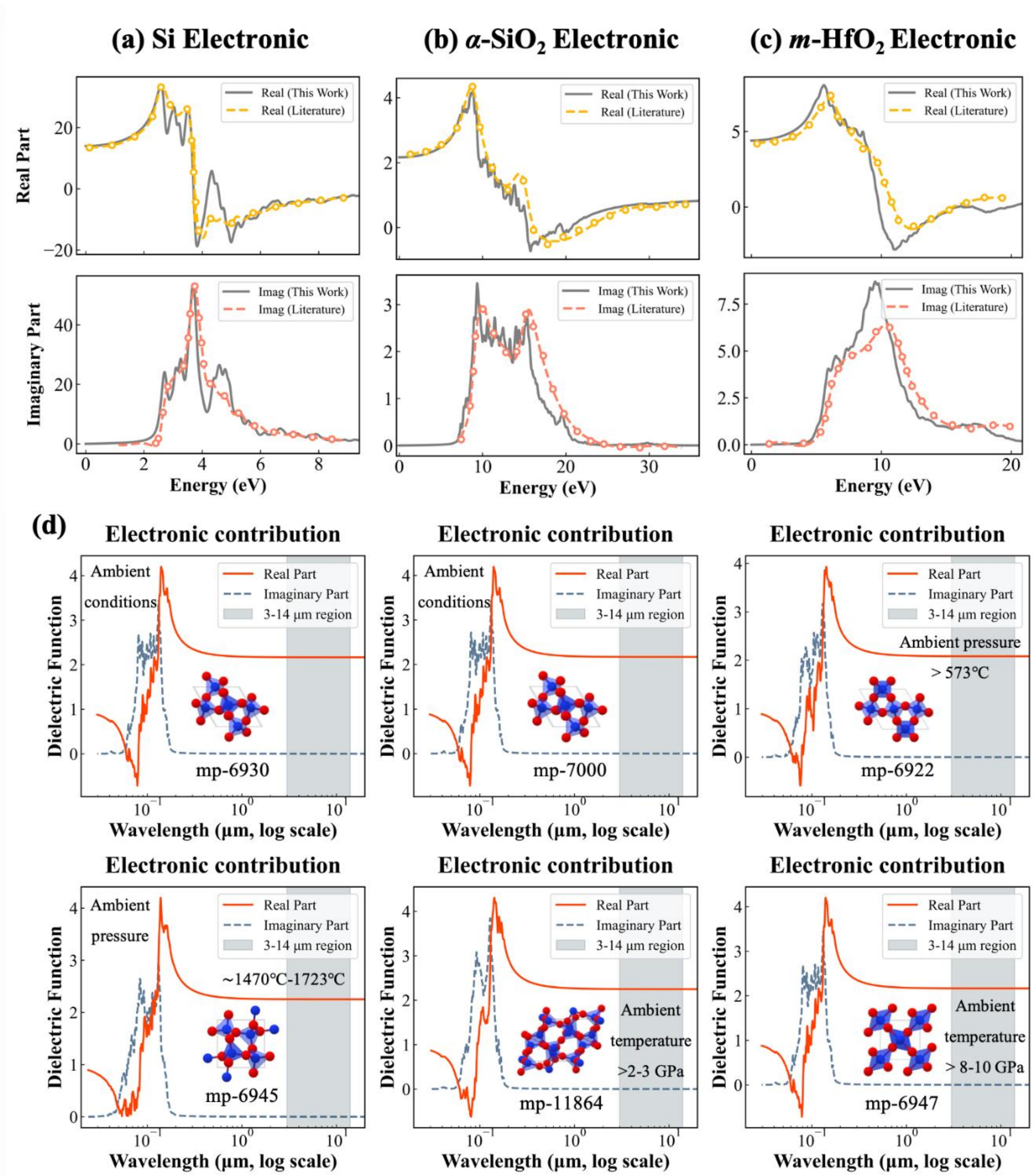


**FIGURE S2** | (a-c) Calculated dielectric functions of Si [3], $SiO_2$ [4], and $HfO_2$ [5] compared with literature data (top: real part; bottom: imaginary part). (d) Dielectric functions of six common and stable $SiO_2$ polymorphs, showing highly similar results with only minor differences in fine spectral features.

To validate the accuracy of the calculated electronic contributions to the dielectric constant, the computed results were systematically compared with literature data. For single-crystal silicon (Si), the theoretical work by Lachenani et al. published in the Bulletin of Materials Science was used as a

benchmark [3]. That study calculated the dielectric function of bulk silicon using a plane-wave pseudopotential method with the GGA-PBE functional. The present results show good agreement with that literature data in the visible to near-infrared region, as shown in **Figure S2(a)**, confirming the reliability of our computational approach.

For silicon dioxide ($SiO_2$), the first-principles study by Khattari et al. was selected for cross-validation [4]. Using the CASTEP package, that work systematically calculated the optical properties of the *α*-quartz structure under hydrostatic pressures of 0-12.5 GPa, reporting a static refractive index of $n = 2.04$ at ambient pressure and a peak of the real part of the dielectric function in the extreme ultraviolet region around 100 nm. The deviation between our calculated values and the literature benchmark is small [**Figure S2(b)**], confirming the suitability of our model.

For hafnium dioxide ($HfO_2$), reference was made to the comprehensive DFT study by Mazumder et al. [5]. That work employed the meta-GGA SCAN (Strongly constrained and appropriately normed) functional to systematically calculate the dielectric properties of monoclinic $HfO_2$ (space group $P2_1/c$). For the electronic contribution to the dielectric constant, the peak positions in the imaginary part of the dielectric function at the key optical transition energy (∼10 eV) are in excellent agreement with the literature data, further validating the reasonableness of our results [**Figure S2(c)**].

In addition, we calculated the dielectric functions of all six stable $SiO_2$ polymorphs (*α*-quartz, *β*-quartz, *α*-cristobalite, coesite and stishovite). The results, shown in **Figure S2(d)**, reveal that the dielectric functions are highly similar overall, with only minor differences in the fine structure. These small variations arise from subtle changes in lattice symmetry and ionic polarisability, but do not alter the overall macroscopic dielectric response trends.

## Supplementary Note 4 | Calculation of optical constants from the frequency dependent dielectric function

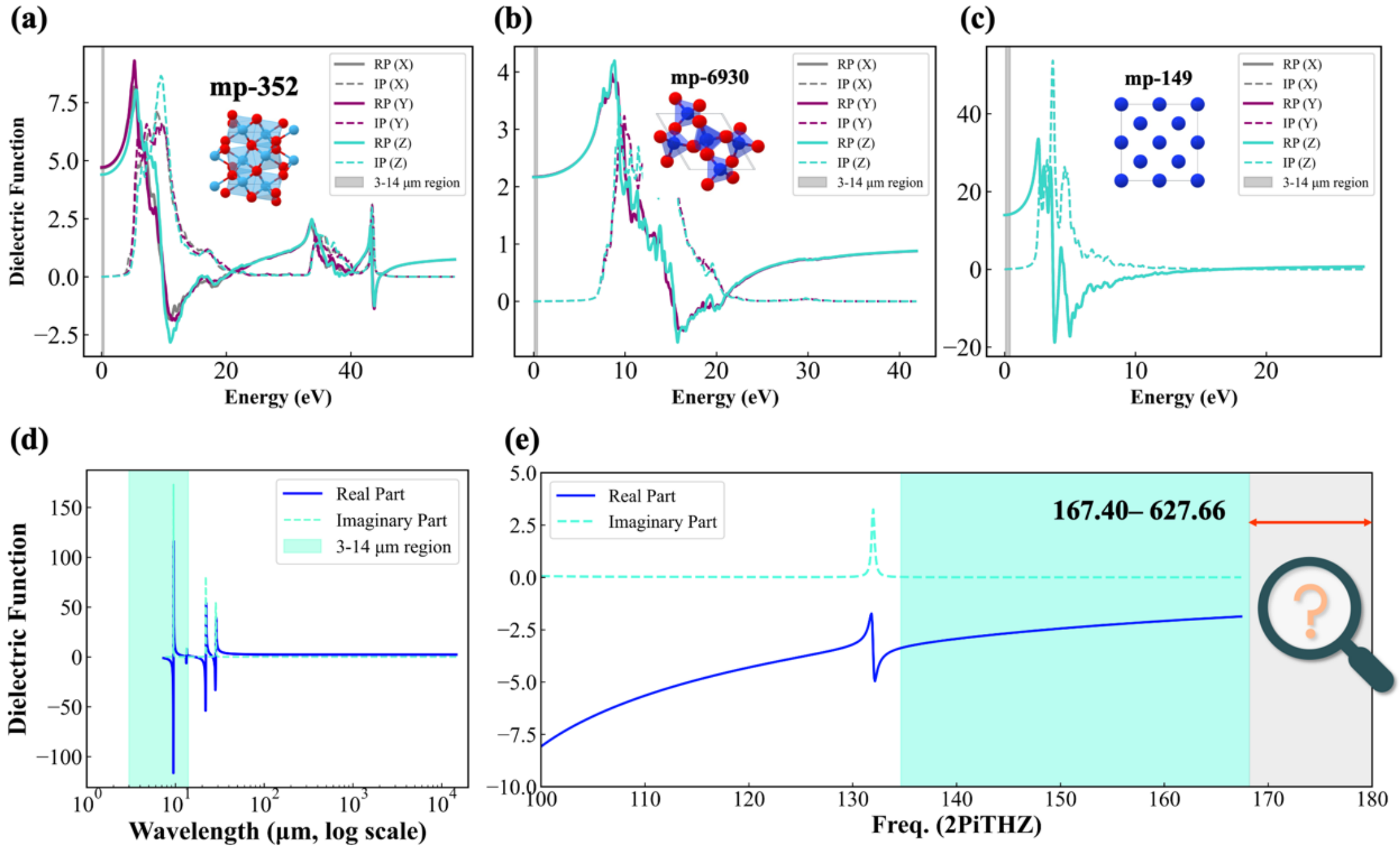


**FIGURE S3** | (a-c) Electronic dielectric function of $HfO_2$, $SiO_2$, and Si, with Si being isotropic and all three anisotropic components shown for $HfO_2$ and $SiO_2$ (directionally averaged for subsequent conversion to optical constants), gray areas mark the 3-14 μm range. (d-e) Ionic dielectric function of $SiO_2$ and $HfO_2$, paleturquoise areas indicate the 3-14 μm spectral region.

The optical dielectric function describes the polarisation response of a material to an electromagnetic field as a function of frequency and is a complex function: $\varepsilon(\omega) = \varepsilon'(\omega) + i\varepsilon''(\omega)$. The real part $\varepsilon'(\omega)$ characterises the material's ability to store electric energy (refractive behaviour), while the imaginary part $\varepsilon''(\omega)$ accounts for energy absorption (loss), i.e., the ability to absorb light. The dielectric function can be interconverted with the refractive index $n$ and the extinction coefficient $k$ via the following relations:

$$\begin{cases} \varepsilon' = n^2 - k^2 \\ \varepsilon'' = 2nk \\ n = \sqrt{\frac{1}{2}\left(\varepsilon' + \sqrt{(\varepsilon')^2 + (\varepsilon'')^2}\right)} \\ k = \sqrt{\frac{1}{2}\left(-\varepsilon' + \sqrt{(\varepsilon')^2 + (\varepsilon'')^2}\right)} \end{cases} \quad \text{(S1)}$$

These transformations apply to linear, homogeneous, isotropic and non-magnetic media (i.e., relative permeability $\tilde{\mu} \approx 1$). Under this condition, the real part $\varepsilon'(\omega)$ is primarily related to the polarisation

strength and the phase delay of light waves, whereas the imaginary part $\varepsilon''(\omega)$ directly quantifies the energy loss due to light absorption. Together, they provide a complete foundation for describing the optical response of a material.

Using the above expressions, the frequency-dependent dielectric functions obtained in the previous chapter can be converted into frequency-dependent optical functions. Because this study focuses on spectral control in the infrared region (3-14 μm), this spectral range is highlighted on the dielectric function plots: grey regions in **Figure S3(a-c)** for the electronic contribution, and paleturquoise regions in **Figure S3(d-e)** for the ionic contribution.

A particular issue arises for $HfO_2$. As noted in the previous chapter, in the target band of 3-14 μm (714-3333 $cm^{-1}$), the DFPT calculation yields a data gap [grey region in **Figure S3(e)**]. This gap originates from a physical boundary of the computational method: VASP solves the ionic dielectric function by integration over discrete phonon frequency points. When the frequency exceeds the maximum phonon frequency $\omega_{\mathrm{max}}$ of the system, the phonon density of states becomes zero, causing the formula's denominator to diverge and no valid solution can be obtained.

Importantly, this computational breakdown actually mirrors a real physical process - in the high-frequency region beyond the phonon resonance frequencies, the ionic polarisation contribution should smoothly decay to zero. However, conventional DFPT cannot describe this decay trajectory, leaving a gap in the dielectric function data in a critical spectral range and consequently affecting the accuracy of subsequent photonic crystal designs.

## Supplementary Note 5 | Analysis of machine learning model extrapolation behaviour

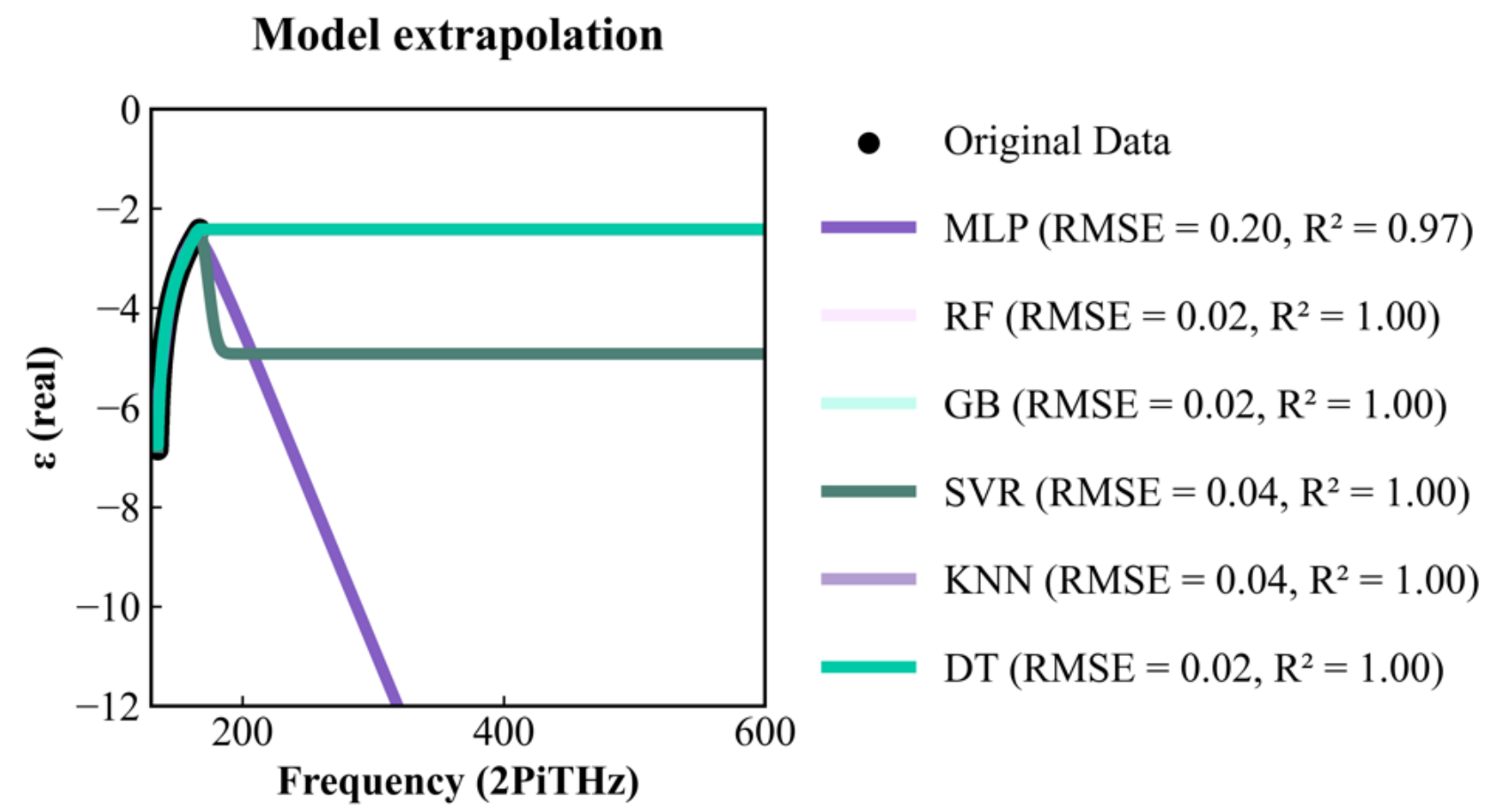


**FIGURE S4** | Extrapolation behaviour of various regression models for the ionic dielectric function of *m*-$HfO_2$ beyond the training frequency range.

All six regression models achieve excellent accuracy on the train and test set within the original frequency range (135.00-167.40 $2\pi \cdot$ THz), demonstrating their strong capacity to fit the dielectric function data where training points exist. The RMSE values are below 0.2 and $R^2$ scores exceed 0.99 for most models, indicating near-perfect interpolation performance. However, as shown in the extrapolation plot (frequency up to 600 $2\pi \cdot$ THz and conceptually beyond), every model fails dramatically when predicting outside the training domain. The physical expectation beyond the maximum phonon frequency (825 $cm^{-1}$ for *m*-$HfO_2$, corresponding to approximately 167.40 $2\pi \cdot$ THz in the code's unit) is that the ionic contribution to the real part of the dielectric function should smoothly decay to zero. Instead, all models produce unphysical oscillations, divergence, or flat trends that do not respect this asymptotic behaviour.

## Supplementary Note 6 | Extrapolation behaviour of six analytical functions for the ionic dielectric function of *m*-$HfO_2$ beyond the training frequency range

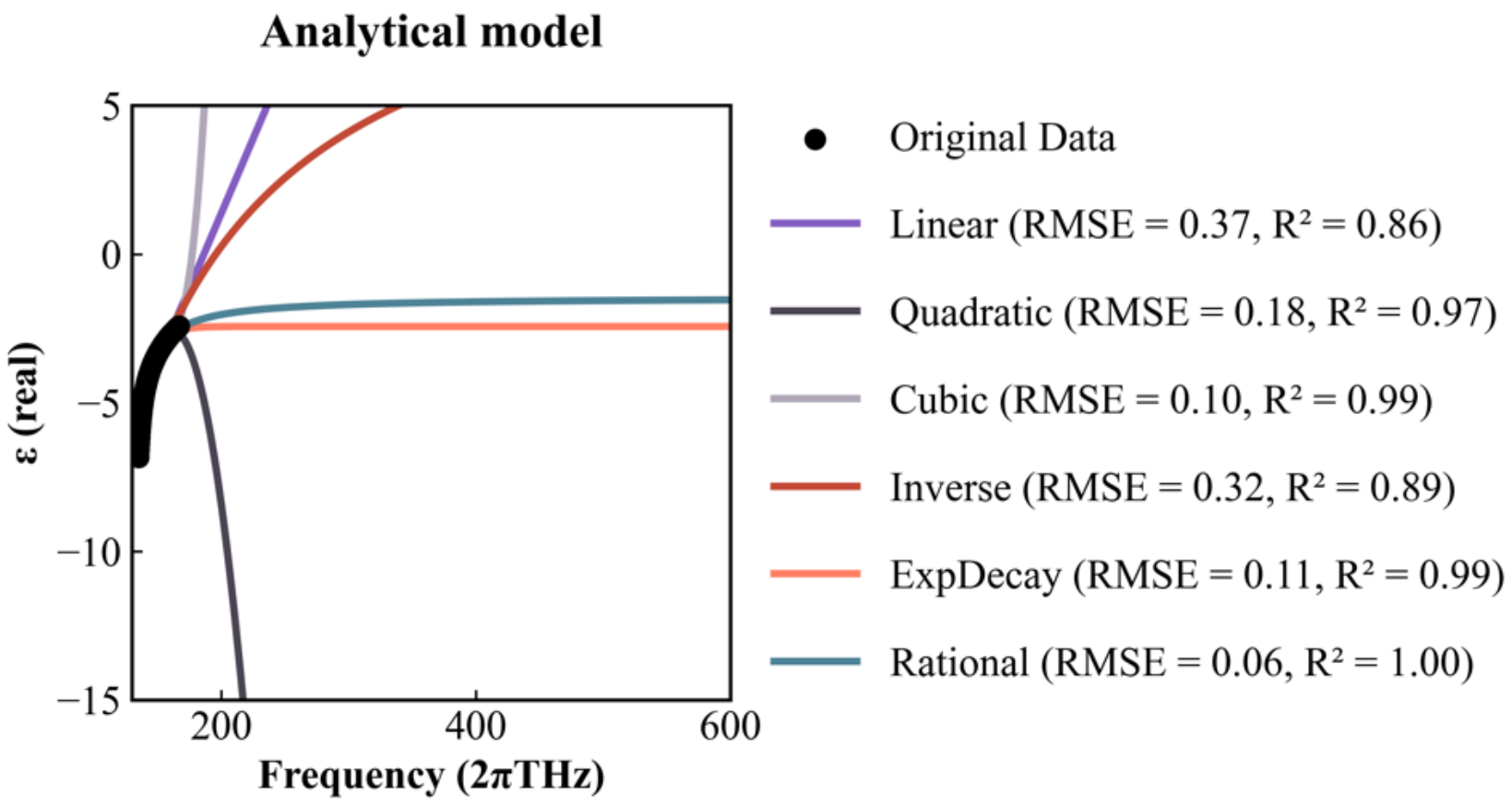


**FIGURE S5** | Extrapolation behaviour of six analytical functions for the ionic dielectric function of *m*-$HfO_2$ beyond the training frequency range.

We tested six analytical functions (linear, quadratic, cubic, inverse, exponential decay, and rational in linear fractional form) to fit the real part of the ionic dielectric function of *m*-$HfO_2$ within the training frequency range (135.00-167.40 $2\pi \cdot$ THz). Their interpolation performance on the training set (see **Figure S5**) is generally poor compared with the machine learning models shown in **Figure S4**, as reflected by significantly higher RMSE and lower $R^2$ values. However, their extrapolation behaviour beyond the training domain is notably better than that of purely data-driven models: most analytical functions do not produce the abrupt divergence or flat plateaus observed in the ML extrapolations. Nevertheless, with the exception of the rational and exponential decay models, all other analytical functions violate basic physical expectations. Physically, at frequencies far above the maximum phonon frequency, the ionic polarisation cannot follow the rapidly oscillating field. Consequently, the ionic contribution to the real permittivity should asymptotically approach zero. While the rational and exponential decay models do exhibit a decaying trend, **Figure S5** shows that they converge to approximately -1.44 instead of zero. For instance, the rational fit yields the expression

$$\varepsilon = \frac{-127x + 1.23 \times 10^4}{88x - 1.11 \times 10^4} \tag{S2}$$

which tends to -127/88 ≈ -1.44 as $x \to \infty$. This negative offset is physically unjustified because the ionic contribution must decay to zero, not to a negative constant. Therefore, neither a simple analytical function nor a conventional machine learning model alone can provide a reliable extrapolation. A hybrid approach that combines a semi-Lorentzian oscillator model (which encodes the known resonance physics) with a machine-learning correction is necessary to achieve both physical consistency and accurate description of the ionic response in the target infrared band.

## Supplementary Note 7 | PRML fitting and extrapolation performance

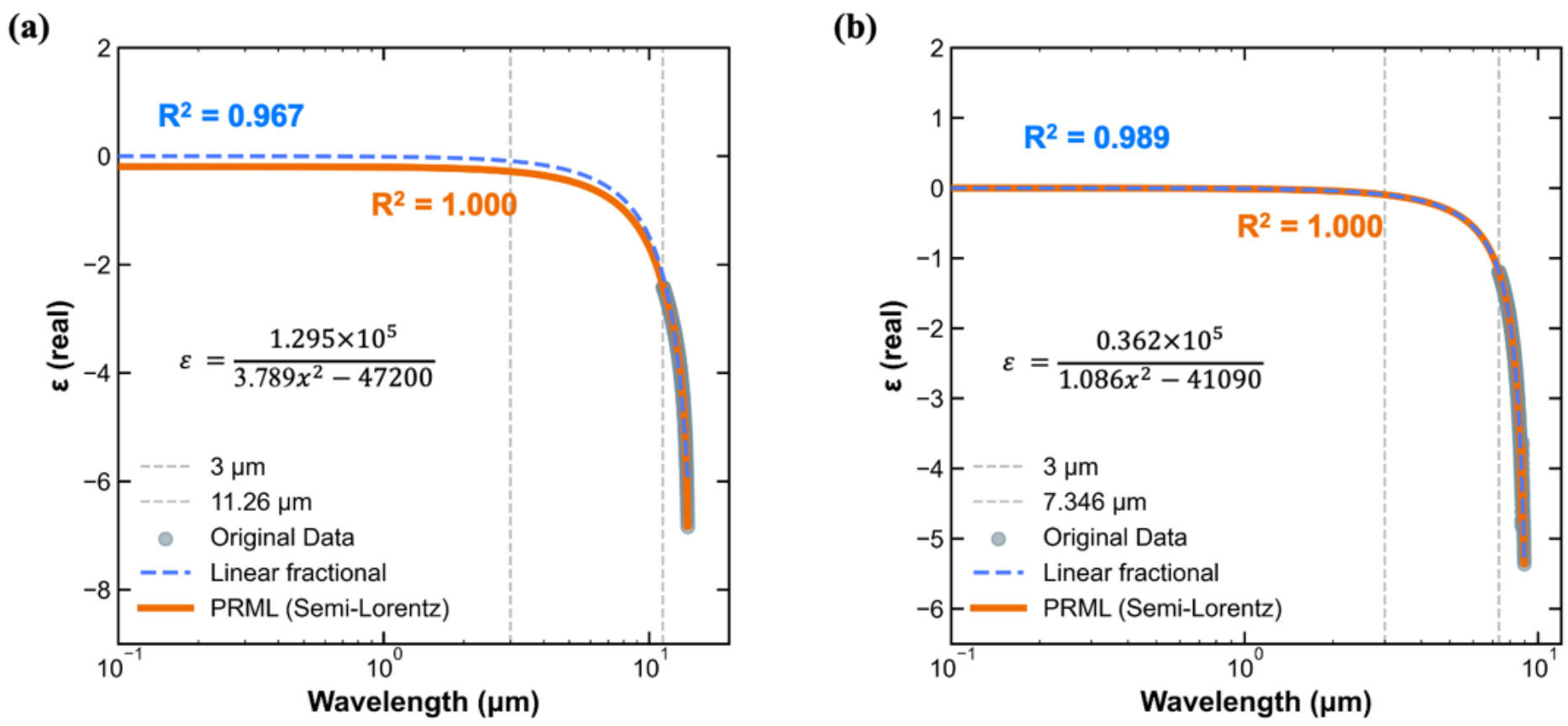


**FIGURE S6** | Predictive performance and extrapolation capability of the PRML model on $HfO_2$ and $SiO_2$. (a) $HfO_2$: comparison of predictions between the PRML model and the linear fractional function within the 11.26-14 μm range, along with the model's extrapolation behavior over the 3-11.26 μm region. (b) $SiO_2$: similar comparison within the 7.346-14 μm range and extrapolation over the 3-7.346 μm region. Overall, the PRML model achieves a fitting accuracy comparable to that of the machine learning model shown in **Figure 3(b)** of the main text, while also retaining the robust extrapolation capability characteristic of analytical models, yielding reliable predictions well beyond the training spectral intervals.

## Supplementary Note 8 | Experimental validation of PRML extrapolation for $HfO_2$

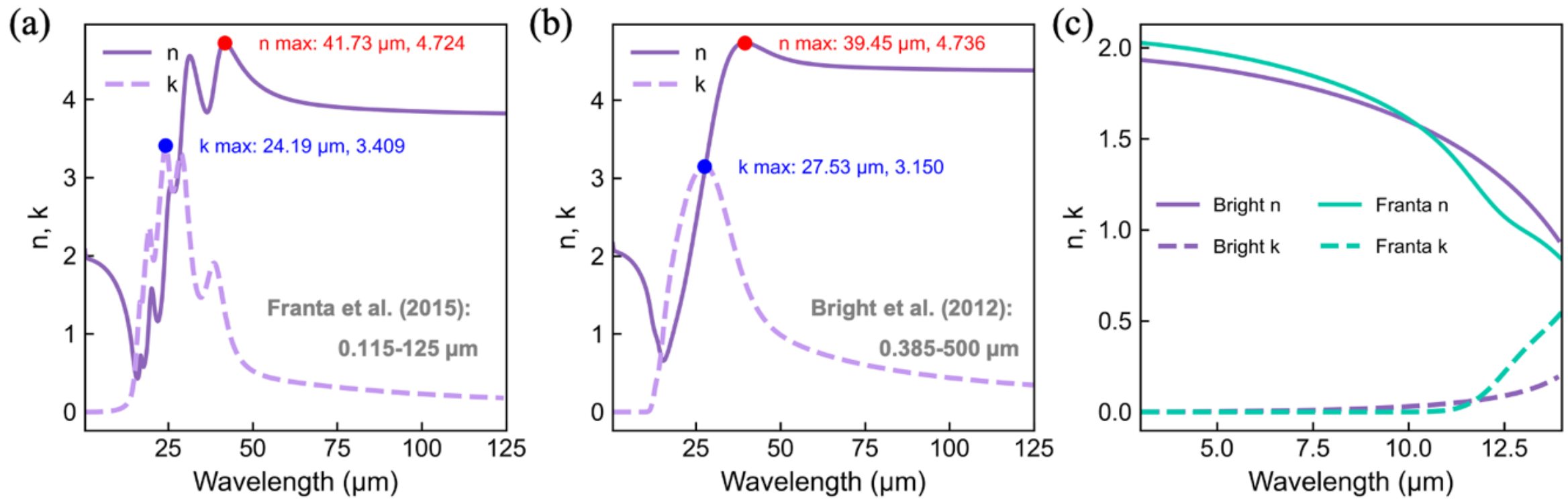


**FIGURE S7** | Experimental characterization and comparative visualization of optical constants ($n$, $k$) of $HfO_2$ across a broad spectral range. (a) Experimental optical constants (refractive index $n$ and extinction coefficient $k$) of $HfO_2$ measured by Franta et al. [6] in the spectral range of 0.115-125 μm. (b) Experimental results reported by Bright et al. [7] in the range of 0.385-500 μm. Panels (a) and (b) are both truncated to the common spectral range of 0.385-125 μm for unified visualization. (c) Visualization and comparison of the optical constants obtained from these two experimental datasets within the specific target band of 3-14 μm focused in this study.

To verify the reliability of the PRML model predictions for the optical constants of $HfO_2$ in the 3-11.26 μm wavelength range, we conducted a quantitative comparison between the model predictions and experimental spectral data from two previously published studies.

**Figure S7(a)** presents the experimental results reported by Franta et al. [6]. In their work, $HfO_2$ thin films with a thickness of approximately 113 nm were prepared via vacuum thermal evaporation. By combining multiple ellipsometers and spectrophotometers, they acquired ellipsometric parameters and transmittance/reflectance spectra covering the spectral range from vacuum ultraviolet (VUV) to far-infrared (FIR). A comprehensive structural model incorporating surface roughness and interfacial transition layers was introduced to perform global fitting, yielding optical constants with a precision on the order of $10^{-3}$ over a broad spectral band.

**Figure S7(b)** illustrates the experimental results from Bright et al. [7]. They deposited $HfO_2$ films with thicknesses ranging from 180 nm to 500 nm using direct current reactive magnetron sputtering at a substrate temperature of 300 ℃. Spectroscopic ellipsometry and Fourier-transform infrared (FTIR) spectroscopy were employed for room-temperature measurements. The experimental data were subsequently analyzed and fitted using the Cauchy dispersion formula combined with a Lorentz multi-oscillator model, enabling the extraction of optical constants covering an ultra-wide spectral range from 0.385 μm to 500 μm.

We further performed a cross-comparison of the optical constants obtained from these two independent experimental datasets within the target band of 3-14 μm. The comparison reveals that, owing to differences in thin-film deposition techniques, measurement equipment, and data fitting models, certain numerical discrepancies exist between the two datasets. Nevertheless, these deviations remain within an acceptable range, and the positions as well as the relative trends of the characteristic absorption peaks are in good agreement, thereby validating the reliability of the experimental data. This provides a robust benchmark for the subsequent optimization designs using the PRML model within the target spectral range.

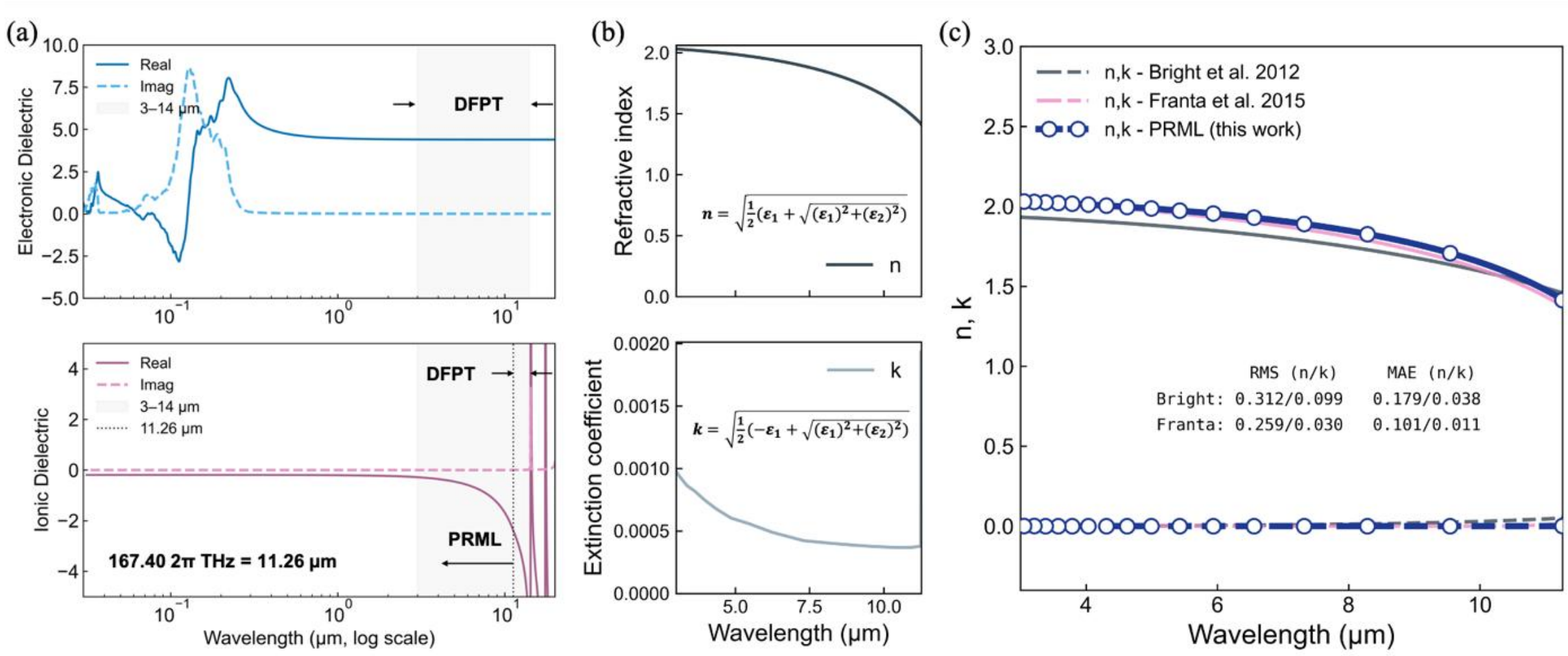


**FIGURE S8** | Theoretical calculations and experimental cross-validation of the broadband dielectric function and optical constants of $HfO_2$. (a) Decomposition of the dielectric function of $HfO_2$ and the broadband spectral stitching. The top panel illustrates the electronic contribution to the dielectric function. The bottom panel illustrates the ionic contribution, where the DFPT-computed band (11.26-14 μm) is smoothly stitched with the PRML-predicted band (3-11.26 μm). (b) Visualization of the complex optical constants (refractive index *n* and extinction coefficient *k*) derived from the total dielectric function within the target band of 3-11.26 μm. (c) Quantitative comparison and validation of the DFPT+PRML combined results against two independent experimental datasets (Bright et al. [7] and Franta et al. [6]) in the 3-11.26 μm band.

To verify the reliability of the PRML model within the 3-11.26 μm band, we introduce the mean absolute error (MAE) as the evaluation metric. For the refractive index *n*, the MAE values are 0.179 compared to the Bright data and 0.101 compared to the Franta data, while for the extinction coefficient *k*, they are 0.038 and 0.011, respectively. Such remarkably low errors fully substantiate the excellent generalization capability and predictive accuracy of the PRML model within the target infrared band, providing a reliable physical foundation for subsequent optical optimization designs.

## Supplementary Note 9 | Theoretical basis for phonon broadening and anisotropy handling in monoclinic $HfO_2$

In standard DFPT frameworks, the adiabatic and harmonic approximations are typically employed. Under the harmonic approximation, phonons are regarded as independent harmonic oscillators with infinite lifetimes, which leads to ideal Dirac delta functions (i.e., infinitely sharp peaks) in the imaginary part of the dielectric function at the phonon frequencies, while the real part undergoes a discontinuous jump at these frequencies.

In actual material systems, however, various relaxation mechanisms - such as phonon–phonon scattering, phonon-electron scattering, as well as impurities or defects - endow phonons with finite lifetimes and introduce noticeable damping effects. According to the energy-time form of the Heisenberg uncertainty principle:

$$\Delta E \cdot \tau \sim \hbar \tag{S3}$$

where $\tau$ denotes the phonon lifetime, characterizing the temporal uncertainty of the phonon's existence. By substituting the relation between phonon energy and frequency, $E = \hbar\omega$ , into the above equation, we obtain:

$$(\hbar \Delta\omega) \cdot \tau \sim \hbar \quad \rightarrow \quad \Delta\omega \cdot \tau \sim 1 \quad \rightarrow \Delta\omega \sim \frac{1}{\tau} \tag{S4}$$

This relationship clearly demonstrates that a shorter phonon lifetime results in a larger frequency broadening $\Delta\omega$. To bridge the gap between theoretical calculations and experimental measurements, the Lorentz oscillator model is conventionally introduced. The essence of this model is to correct the idealized assumption of infinite phonon lifetimes in DFPT by incorporating the damping effect caused by finite phonon lifetimes. Upon introducing the damping coefficient $\gamma$, the complete expression for the complex dielectric function $\varepsilon(\omega)$ is given by:

$$\varepsilon(\omega) = \varepsilon_\infty + \sum_j \frac{S_j}{\omega_0^2 - \omega^2 - i\omega\gamma_j} \tag{S5}$$

The corresponding real and imaginary parts are expressed as follows:

$$\varepsilon'(\omega) = \varepsilon_\infty + \sum_j \frac{S_j\left(\omega_j^2 - \omega^2\right)}{\left(\omega_j^2 - \omega^2\right)^2 + \left(\gamma_j\omega\right)^2} \tag{S6}$$

$$\varepsilon''(\omega) = \sum_j \frac{S_j \cdot \gamma_j\omega}{\left(\omega_j^2 - \omega^2\right)^2 + \left(\gamma_j\omega\right)^2} \tag{S7}$$

where $\omega_j$ and $S_j$ are directly derived from DFPT calculations, and $\gamma_j$ is the introduced damping coefficient (i.e., the broadening parameter), which may differ for different phonon modes. The subscript $j$ denotes the indices of individual phonon modes. The macroscopic dielectric response of the material is given by the linear superposition of contributions from all phonon modes, thereby necessitating the simultaneous broadening treatment of multiple peaks.

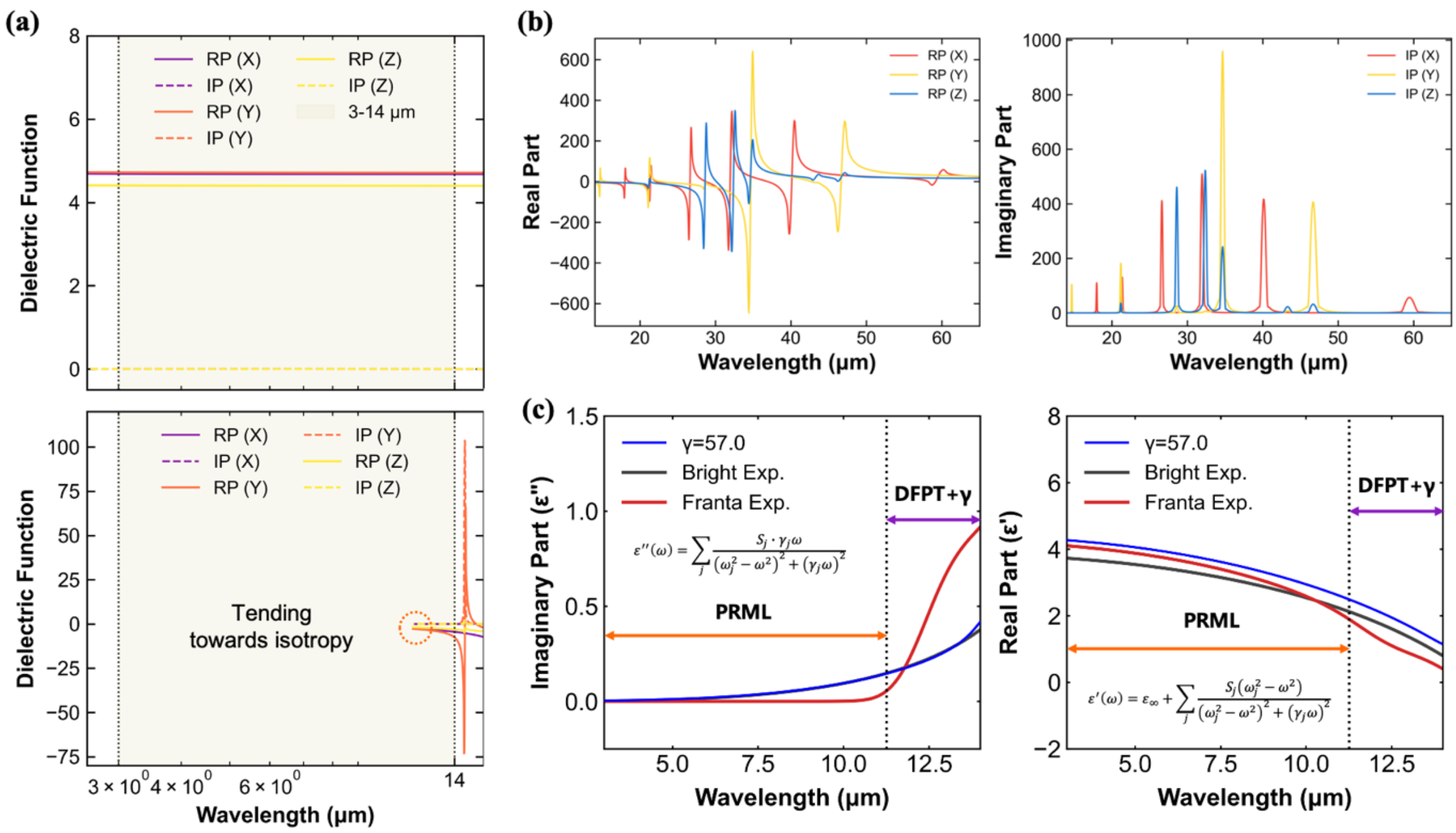


**FIGURE S9** | Dielectric functions of $HfO_2$ and oscillator model validation. (a) Dielectric functions over 3-14 μm (upper: electronic; lower: ionic). The ionic parts show decreasing values with wavelength for three crystallographic directions, with real parts converging at long wavelengths. (b) Real and imaginary ionic dielectric functions over 14-65 μm, exhibiting sharp resonance peaks at distinct wavelengths for the three directions, indicating anisotropic phonon modes. (c) Comparison of broadening parameters from Lorentz oscillator fitting with experimental data [6, 7], showing good agreement.

Before discussing the results, it is necessary to clarify the anisotropy issue of $HfO_2$. As a material with a monoclinic structure, $HfO_2$ exhibits significant differences in both phonon frequencies and oscillator strengths along different lattice directions. However, within the 3-14 μm band targeted in this study, as the wavelength decreases, the electronic contribution becomes nearly completely isotropic, and the ionic contribution also gradually tends toward isotropy; at the wavelength of 11.26 μm, the values in all directions are almost equal, see **Figure S9(a)**. To simplify the treatment, when performing PRML fitting in the 3-11.26 μm band, we adopt the isotropic effective medium approximation. Specifically, we define the theoretical polycrystalline dielectric constant by taking the arithmetic mean of the three diagonal elements of the dielectric tensor (*xx*, *yy*, *zz*):

$$\varepsilon_{\text{iso}}(\omega) = \frac{\varepsilon_{xx}(\omega) + \varepsilon_{yy}(\omega) + \varepsilon_{zz}(\omega)}{3} \tag{S8}$$

Based on this simplified scheme, the mean squared errors (MSE) for the PRML-predicted refractive index $n$ and extinction coefficient $k$ within the 3-11.26 μm band are 0.024 and 0.001, respectively, which fully validates the reasonableness and reliability of this approximation.

Nevertheless, for the wavelength range beyond 11.26 μm, the peak positions of the three directions show significant divergence, see **Figure S9(b)**. Along the increasing wavelength direction, the first peak of the *xx* direction appears at 17.98 μm, whereas the first peaks for the *yy* and *zz* directions are located at 14.68 μm; within the 3-11.26 μm range, none of these directions exhibit any peaks. Given that experimentally deposited thin films typically possess a preferred crystallographic orientation, directly applying the mathematical average of the three directions would inevitably lead to severe mismatch between the theoretical model and experimental observations. Therefore, referring to the work of Bright et al., the as-prepared $HfO_2$ films clearly exhibit a preferred orientation rather than a completely random isotropic distribution. Accordingly, when comparing theoretical calculations with experimental spectra, we adopt the theoretical results along the *xx* direction for broadening fitting.

Finally, the determination of the only adjustable parameter in the formula - the damping coefficient (broadening parameter) - is briefly addressed. The physical origin of the damping factor lies in the finite lifetime of optical phonons, which can be directly extracted via ab initio molecular dynamics (AIMD) simulations. Although Bright et al. [7] numerically obtained the broadening (damping factor) through first-principles calculations, this is not the primary focus of the present study, and thus will not be elaborated upon in detail here.

## Supplementary Note 10 | Visualization of FM hyperparameter optimization with specified parameters

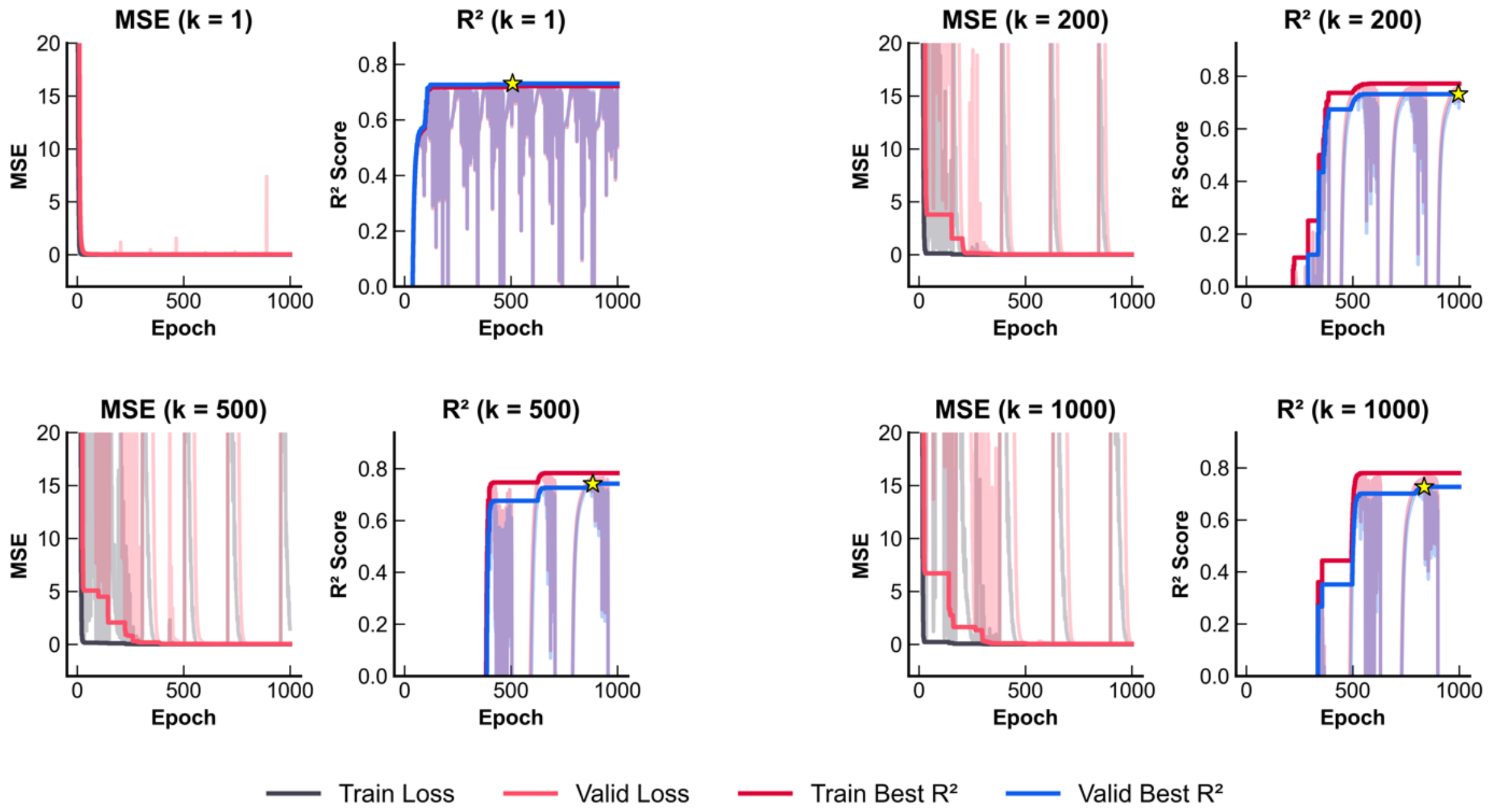


**FIGURE S10** | Training iteration curves of FM models ($k$ = 1, 200, 500, 1000). Left panel: mean squared error (MSE) versus iterations; right panel: $R^2$ versus iterations. Four curves correspond to $k$ = 1, 200, 500, and 1000, respectively.

## Supplementary Note 11 | Small sample driven metamaterial design breaking classical optimization limits

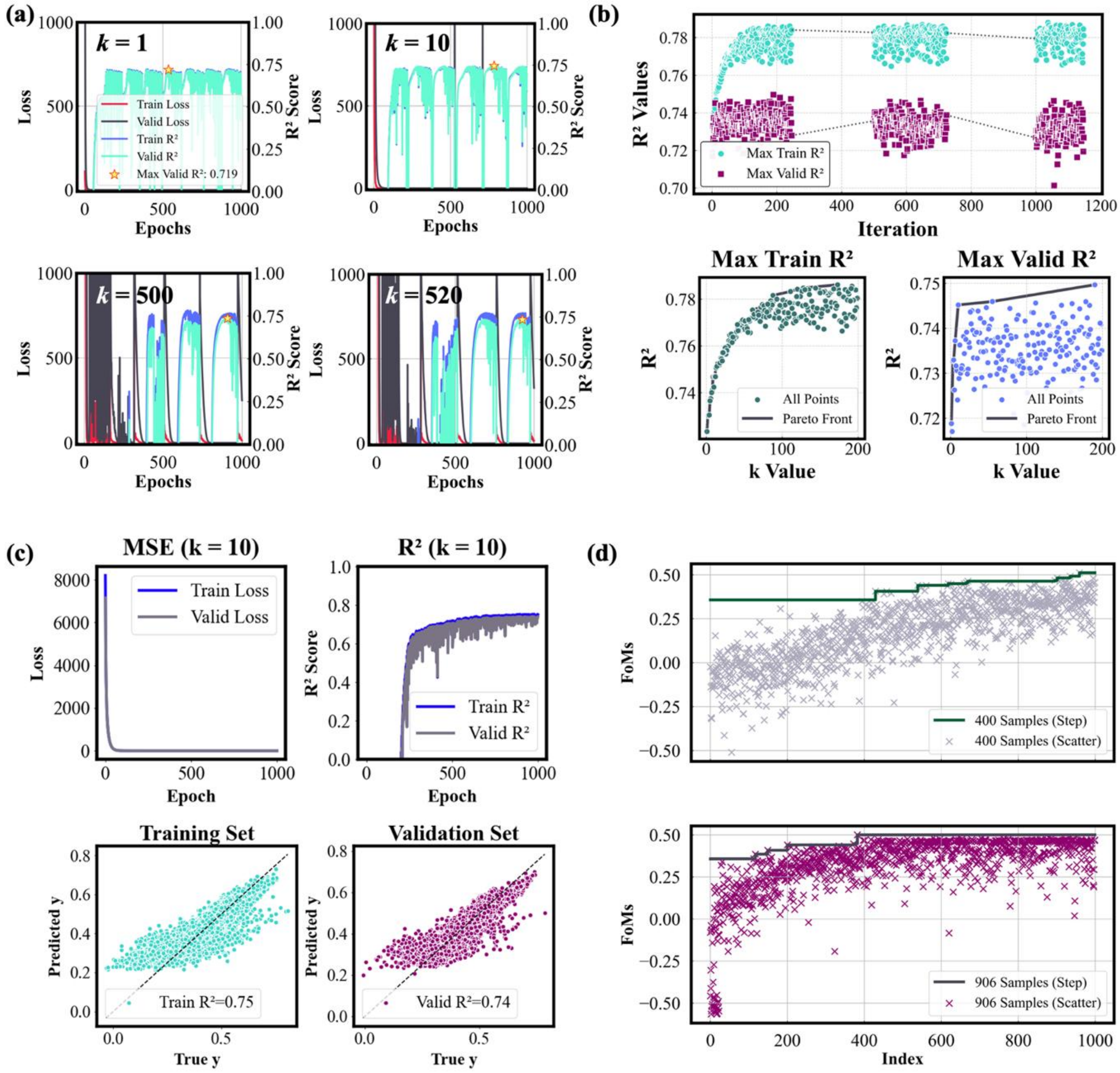


**FIGURE S11** | FM hyperparameter optimization and annealer performance evaluation. (a) Training curves under different *k*. (b) Hyperparameter performance comparison and Pareto front. (c) Training convergence and scatter plots at *k* = 10. (d) Annealer optimization process comparison on datasets of different sizes.

## Supplementary Note 12 | Performance comparison and high-dimensional design space distribution between quantum-inspired Ising sampling and Monte Carlo search

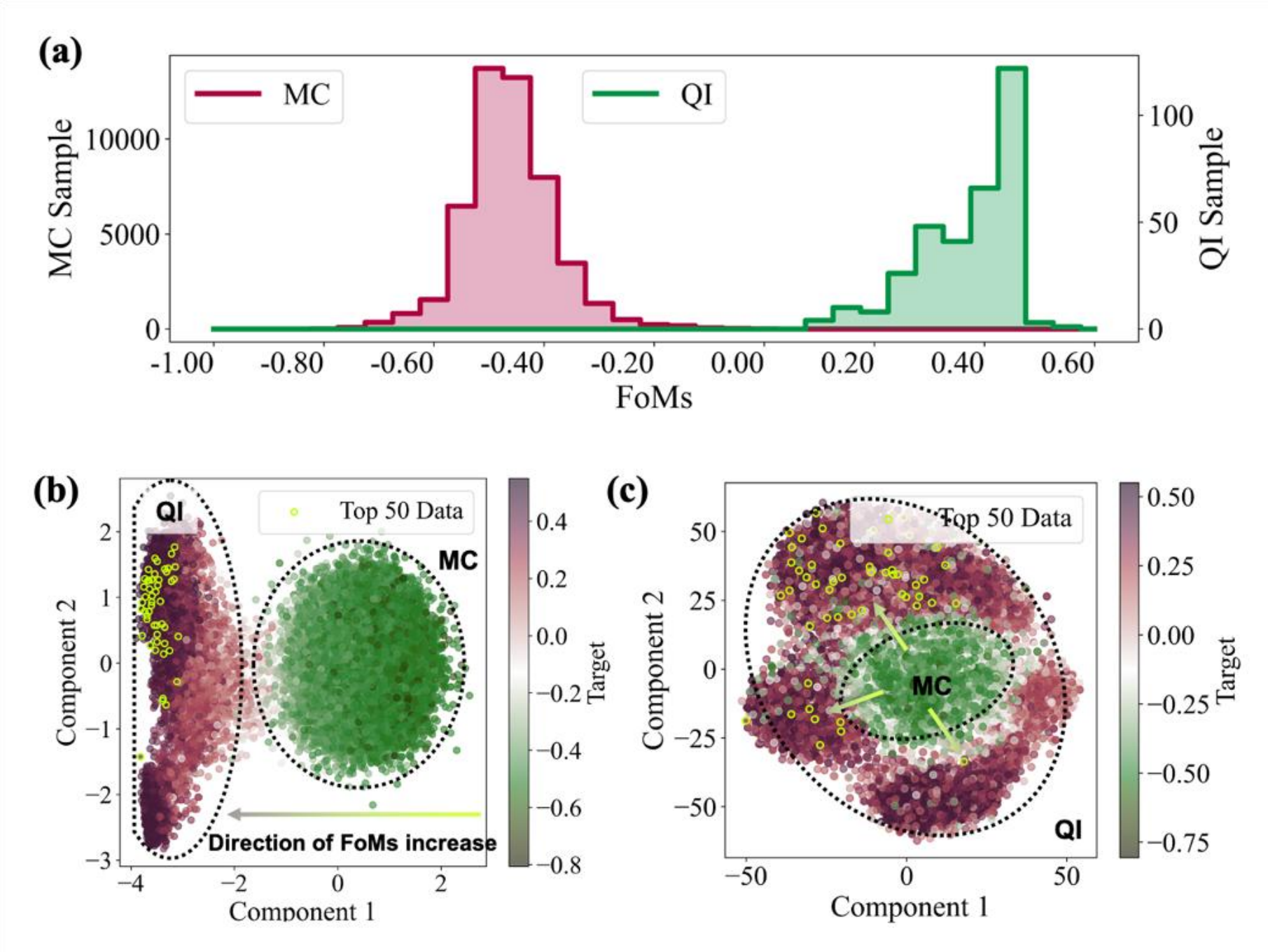


**FIGURE S12** | Performance comparison and high-dimensional design space distribution between quantum-inspired Ising sampling and Monte Carlo random search. (a) Histogram of FoM distributions: the QI-Annealer yields FoM values predominantly centered around 0.5 over 330 independent optimization runs, whereas 50 thousand Monte Carlo searches produce FoM values mostly concentrated near -0.5, with only very few samples exceeding FoM > 0. (b) PCA visualization: QI-sampled points cluster into a narrow vertical strip on the left side of the projection space, while MC samples form a dense circular cluster on the right. The top-50 high-FoM samples (highlighted in fluorescent green) almost exclusively reside within the QI cluster, confirming that the high-performance region is effectively covered only by QI sampling. (c) t-SNE visualization: MC samples appear as a dense annular cluster at the center, whereas QI samples extend outward from this center along a specific search trajectory. Together, these two unsupervised dimensionality reduction analyses demonstrate that quantum-inspired optimization, under a very limited sampling budget, can effectively locate high-performance regions of the solution space that conventional uniform random search struggles to reach, exhibiting markedly superior directed exploration efficiency over large-scale Monte Carlo random search.

## References for Supporting Information


1. M. K. Horton, P. Huck, R. X. Yang, et al., "Accelerated Data-Driven Materials Science with the Materials Project," *Nature Materials* 24, no. 10 (2025): 1522, https://doi.org/10.1038/s41563-025-02272-0.
2. A. Jain, S. P. Ong, G. Hautier, et al., "Commentary: The Materials Project: A Materials Genome Approach to Accelerating Materials Innovation," *APL Materials* 1, no. 1 (2013): 011002, https://doi.org/10.1063/1.4812323.
3. H. Lachenani, S. Ouir, F. Boudeffar, et al., "Study of Optical Properties of Porous Silicon by DFT, Comparison to Experimental and Effective Medium Approximation Methods," *Bulletin of Materials Science* 47, no. 3 (2024): 128, https://doi.org/10.1007/s12034-024-03189-1.
4. Z. Y. Khattari, S. Al-Omari, F. Afaneh, "Optical and Electrical Properties of $SiO_2$ under Uniaxial, Biaxial, and Isotropic Pressure: a First Principles Study," *Silicon* 16, no. 11 (2024): 4713, https://doi.org/10.1007/s12633-024-03038-0.
5. J. T. Mazumder, R. Mayengbam, S. K. Tripathy, "Theoretical Investigation on Structural, Electronic, Optical and Elastic Properties of $TiO_2$, $SnO_2$, $ZrO_2$ and $HfO_2$ Using SCAN Meta-GGA Functional: A DFT Study," *Materials Chemistry and Physics* 254, (2020): 123474, https://doi.org/10.1016/j.matchemphys.2020.123474.
6. D. Franta, D. Nečas, I. Ohlídal, "Universal Dispersion Model for Characterization of Optical Thin Films over a Wide Spectral Range: Application to Hafnia," *Applied Optics* 54, no. 31 (2015): 9108, https://doi.org/10.1364/AO.54.009108.
7. T. J. Bright, J. I. Watjen, Z. Zhang, C. Muratore, A. A. Voevodin, "Optical Properties of $HfO_2$ Thin Films Deposited by Magnetron Sputtering: From the Visible to the Far-infrared," *Thin Solid Films* 520, no. 22 (2012): 6793, https://doi.org/10.1016/j.tsf.2012.07.037.
